# Dissecting the heterogeneity and tumorigenesis of BRCA1 deficient mammary tumors via single cell RNA sequencing

**DOI:** 10.7150/thno.63995

**Published:** 2021-10-25

**Authors:** Heng Sun, Jianming Zeng, Zhengqiang Miao, Kuan Cheok Lei, Chen Huang, Lingling Hu, Sek Man Su, Un In Chan, Kai Miao, Xu Zhang, Aiping Zhang, Sen Guo, Si Chen, Ya Meng, Min Deng, Wenhui Hao, Haipeng Lei, Ying Lin, Zhonglin Yang, Dongjiang Tang, Koon Ho Wong, Xiaohua Douglas Zhang, Xiaoling Xu, Chu-Xia Deng

**Affiliations:** 1 Cancer Center, Faculty of Health Sciences, University of Macau, Macau SAR, China.; 2 Centre for Precision Medicine Research and Training, Faculty of Health Sciences, University of Macau, Macau SAR, China.; 3 MOE Frontiers Science Center for Precision Oncology, University of Macau, Macau SAR, China.; 4 Zhuhai Research Institute, University of Macau, China.; 5 Zhuhai Precision Medical Center, Zhuhai People's Hospital, Jinan University, Zhuhai, China.; 6 Breast Disease Center, the First Affiliated Hospital, Sun Yat-Sen University, Guangzhou, China.; 7 Zhuhai SanMed Biotech Ltd, Zhuhai, China.; 8 Joint Research Center of Liquid Biopsy in Guangdong, Hong Kong and Macao, Zhuhai, China.

**Keywords:** *Brca1*/*BRCA1*, single cell RNA-seq, tumor heterogeneity, mammary tumor, *Mrc2*

## Abstract

**Background:** BRCA1 plays critical roles in mammary gland development and mammary tumorigenesis. And loss of BRCA1 induces mammary tumors in a stochastic manner. These tumors present great heterogeneity at both intertumor and intratumor levels.

**Methods:** To comprehensively elucidate the heterogeneity of BRCA1 deficient mammary tumors and the underlying mechanisms for tumor initiation and progression, we conducted bulk and single cell RNA sequencing (scRNA-seq) on both mammary gland cells and mammary tumor cells isolated from *Brca1* knockout mice.

**Results:** We found the BRCA1 deficient tumors could be classified into four subtypes with distinct molecular features and different sensitivities to anti-cancer drugs at the intertumor level. Whereas within the tumors, heterogeneous subgroups were classified mainly due to the different activities of cell proliferation, DNA damage response/repair and epithelial-to-mesenchymal transition (EMT). Besides, we reconstructed the BRCA1 related mammary tumorigenesis to uncover the transcriptomes alterations during this process via pseudo-temporal analysis of the scRNA-seq data. Furthermore, from candidate markers for BRCA1 mutant tumors, we discovered and validated one oncogene *Mrc2*, whose loss could reduce mammary tumor growth *in vitro* and *in vivo*.

**Conclusion:** Our study provides a useful resource for better understanding of mammary tumorigenesis induced by BRCA1 deficiency.

## Introduction

Germline mutations of *BRCA1* predispose women to early onset of breast and ovarian cancers 1. And majority of BRCA1-related breast cancers belong to the most refractory triple negative breast cancer (TNBC) subtype, bearing basal-like feature 2, though the cancers may mainly originate from the luminal mammary epithelia 3, 4. Meanwhile, mutations of *BRCA1* have also been identified in other subtypes of breast cancers 2. Through its diverse functions in DNA damage repair, cell cycle control, transcription regulation, ubiquitination and so on, BRCA1 acts as a very significant tumor suppressor and genomic safeguard 5, 6. BRCA1 deficiency induces severe cellular stress; when occurring in the mammary glands, it impairs the regular developmental process and eventually causes tumorigenesis due to accumulation of genome instability and other alterations 6-9.

We have previously established a mouse model carrying mammary specific knockout of *Brca1* (*MMTV-Cre*;* Brca1^fl/fl^*), which spontaneously develops mammary tumors in a stochastic manner 10. The tumors display variable histopathological features, which could even be detected at different intratumor regions 11. Such inter- and intra-tumor heterogeneity could also be observed in human breast cancers carrying *BRCA1* mutations. According to previous studies, around 40% of BRCA1-related breast cancers belong to ductal carcinoma, not otherwise specified (ductal NOS) type, while typical medullary carcinoma (MC) feature can be observed in 7.8% to 13% of BRCA1-associated breast carcinomas 12-14. More than half of BRCA1-related breast cancers harbor *TP53* mutation and *MYC* amplification 15. Besides, about 78% of breast cancers arising in *BRCA1* mutation carriers are ER-negative, 79% are PR-negative, 90% are HER2-negative, and 69% are Triple-negative 16. Such heterogeneity of histopathology, genetic alterations and gene expression patterns not only reflects the complexity of mammary tumorigenesis induced by BRCA1 deficiency, but also leads to the difficulty of the efficient treatment on such refractory cancers. Meanwhile, more details are still badly lacking to figure out the origin and evolution of the heterogeneity among and within BRCA1-deficient mammary tumors.

scRNA-seq has been widely harnessed to uncover the diverse cell types within heterogeneous cell populations, like many well differentiated organs 17 and many types of cancers 18. In this study, we performed bulk RNA-seq and other experiments to elucidate the intertumor heterogeneity among BRCA1 deficient mammary tumors, and scRNA-seq of more than 20000 mammary cells and tumor cells to reveal the intratumor heterogeneity and gene expression variations during the mammary tumorigenesis process. We also identified and validated one oncogene *Mrc2*, whose loss could block the tumor cell growth both *in vitro* and *in vivo*.

## Materials and methods

### Experimental mice

All animals were housed and handled in accordance with the guidelines of the Institutional Animal Care and Use Committee of the University of Macau. The *MMTV-Cre; Brca1^fl/fl^,* and* MMTV-Cre; Brca1^fl/fl^; Trp53^fl/+^* mice were described previously 10 and were crossed with *ROSA^mT/mG^* mice 19 to generate the *MMTV-Cre; Brca1^fl/fl^; ROSA^mT/mG^, MMTV-Cre; Trp53^fl/+^; ROSA^mT/mG^*, and *MMTV-Cre; Brca1^fl/fl^; Trp53^fl/+^; ROSA^mT/mG^* strains for monitoring the CRE-active cells in mammary gland. Mice were in a C57BL/6 and 129/Sv mixed background. The presence of floxed, deleted *Trp53* and *Brca1* alleles, *mTmG* alleles and of the *Cre* recombinase allele was confirmed by PCR with tail DNA as template 10, 19.

### Cell lines

The immortalized mouse *Brca1*-WT epithelial cell line B477 and *BRCA1*-mutant epithelial cell line G600 were derived from the mammary gland of *Brca1*-WT (*Brca1^+/+^; Trp53^+/-^*) and *Brca1*-mutant (*Brca1^Δ/Δ^;Trp53^+/-^*) mice, respectively 20. Several BRCA1 deficient mouse mammary tumor cell lines were derived from the mammary tumors of the *MMTV-Cre; Brca1^fl/fl^,* or* MMTV-Cre; Brca1^fl/fl^; Trp53^fl/+^* mice. The MCF7, MDA-MB-231, MDA-MB-436 cell lines, and the 293FT cell line for lentiviral production were obtained from American Tissue Culture Collection (ATCC). All cells were cultured in Dulbecco's modified Eagle medium (DMEM) (Gibco Life Technologies) containing 10% FBS with or without 100 IU/mL penicillin, and 100 µg/mL streptomycin.

### Histology and antibody staining

For histology, tissues were fixed in the 10% formalin, blocked in paraffin, sectioned as 5 µm thick, stained with hematoxylin and eosin, and examined by light microscopy. Antibodies against ERalpha (sc-542; Santa Cruz), PR (sc-166169; Santa Cruz), ERBB2 (sc-33684; Santa Cruz), and MRC2 (sc-271148; Santa Cruz) were used for immunohistochemical staining, by using a Histostain® Plus Broad Spectrum kit (859043; Life Technologies) as per the manufacturer's instruction. Antibodies against Keratin14 (PRB-155P; Covance), Keratin18 (sc-53256; Santa Cruz), E-Cadherin (3195; Cell Signaling Technology) and Vimentin (5741; Cell Signaling Technology) were used for immunoflouresencent staining, and nuclei were stained with DAPI (62248; ThermoFisher Scientific).

### Analysis of public human breast cancer data

We reanalyzed molecular data on 6113 breast cancers from previous studies collected in cBioPortal, including data from METABRIC and TCGA, *etc.* 21-28. Among these cancer samples, 169 (2.76%) harbored the genetic alteration on *BRCA1*. While some alterations of *BRCA1*, like amplification, may not be oncogenic as BRCA1 function as a well-known tumor suppressor, others including deep deletion, truncating and missense mutations (putative driver) were considered as *BRCA1* deficient alterations here. Within the 63 *BRCA1* genetically deficient cases, matched RNA-seq data were only available from 13 samples in TCGA database. Meanwhile, 16 TCGA breast cancers with germline pathogenic *BRCA1* mutation 29 were also identified. On these 29 *BRCA1* deficient breast cancers with RNA-seq, we performed the hierarchy clustering analysis based on overall transcriptome and molecular subtyping analysis based on PAM50 according to previous study 30. Besides, a heatmap describing the expression patterns of several mammary basal/luminal lineage markers and epithelial/mesenchymal markers were drawn based on the RNA-seq data by using R 31.

To determine the protein level of MRC2 in human breast tissue and breast cancers, IHC results are cited from human protein atlas (https://www.proteinatlas.org/); and the relative protein levels of MRC2 are collected from Clinical Proteomic Tumor Analysis Consortium (CPTAC) Confirmatory/Discovery dataset 32.

### Bulk tissue RNA sequencing

The mammary tumors from *Brca1*-deficient mice were cut into small pieces and homogenized in TRIzol reagent (15596018; ThermoFisher scientific) by using the Precellys®24 homogenizer (P000669-PR240-A; Bertin Technologies) and tissue homogenizing CKmix (KTO3961-1-009.2; Bertin Technologies) as per the manufacturer's instruction. About 5×10^5^ freshly FACS sorted DAPI^-^CD24^Hi^CD29^Lo^ luminal cells were re-suspended in TRIzol reagent after washing with PBS. After isolated from the TRIzol lysate, the RNA samples were constructed into RNA sequencing libraries by using a NEBNext® Ultra™ RNA Library Prep Kit for Illumina® (E7530L, New England Biolabs) according to the manufacturer's instruction. Sequencing was performed on Illumina HiSeq2500 or HiSeq X Ten platforms by multiplexed paired end run with 100 or 150 cycles.

The RNA sequencing reads were aligned to the reference mm10 mouse genome with TopHat 2.1.1 by using default parameters 33. Gene expression was quantified as FPKM using Cufflinks 2.2.1 34. To cluster the 23 *Brca1*-deficient mouse mammary tumors into subgroups, hierarchical clustering was performed using R with default parameters. Differentially expressed genes among sub-clusters were identified by using DESeq2 35. And, the following cellular function analysis of differentially expressed genes was performed using DAVID 36.

### Single cells suspension preparation

Three-to-four month-old female virgin or pregnant at day 12.5 mice were sacrificed to isolate the fourth pair of mammary glands for single-cell preparation as previously described 37. In brief, the mammary glands were minced, washed in PBS and digested in DMEM/F12 containing 300 U/mL collagenase III (S4M7602S, Worthington, Lakewood, NJ), 100 U/mL hyaluronidase (H3506; Sigma-Aldrich), 5% FBS (Gibco), 5 µg/mL insulin (350-020; Biosource, Rockville, MD), 10 ng/mL EGF (13247-051, Invitrogen), and 500 ng/mL hydrocortisone (H0888; Sigma-Aldrich) for about 1 hour at 37 °C, 5% CO_2_. The resultant organoid suspension was sequentially re-suspended in DMEM/F12 supplemented with 5 mg/mL dispase II (825-001; Roche Diagnostics, Indianapolis, IN) and 0.1 mg/mL DNase I (58C10349; Worthington) for 5 min at 37 °C, and then digested with 0.25% trypsin-EDTA for 1-2 min, and treated with RBC lysis buffer (00433-57; eBioscience, San Diego, CA) for 3 min to remove the red blood cells before filtration through a 40-µm cell strainer (352340, BD Falcon) to obtain single-cell suspension. Enrichment of Lin^-^ epithelial cells was achieved through selective depletion of hematopoietic and endothelial cells by using the EasySeq mouse epithelial cell enrichment kit according to the manufacturer (19758; Stemcell). When the mammary tumors grew to around 1 cm in diameter, they were removed from the *Brca1*-defeicient mice for single-cell preparation as per the same method.

### Fluorescence-activated cell sorting (FACS)

The enriched Lin^-^ mouse mammary epithelial cells and tumor cells were stained at a concentration of 1×10^6^ cells per 100 µl of FACS buffer (HBSS with 0.5% BSA and 2 mM EDTA). Antibodies against mouse CD24 (anti CD24-PE-Cy™7, 560536, BD), and CD29 (anti CD29-APC, 102216, Biolegend) were used to stain the cells. DAPI (1 µg/mL) was also used as an indicator for cell viability. Antibody incubations were performed at 4 °C for 30 min. Then the cells were washed with PBS for twice before sorting by using FACSAria III Cell Sorter (Becton Dickinson). DAPI^-^CD24^Hi^CD29^Lo^mGFP^+^ mammary luminal cells and DAPI^-^CD24^+^ tumor cells were sorted out for following single cell RNA sequencing.

### Fluidigm C1 platform based single cell capture and RNA sequencing

The sorted mammary luminal cells or tumor cells re-suspended in FACS buffer (300-500 cells/µL) were mixed (3:2 ratio) with C1 Cell Suspension Reagent (100-6201, Fluidigm) before loading onto a 10 to 17-µm-diameter C1 Integrated Fluidic Circuit (IFC 100-5760, Fluidigm) 38. Each capture site was carefully examined and recorded under a ZEISS microscope in bright field channel for cell doublets. Cell lysing, reverse transcription and cDNA amplification were performed on the C1 Single-Cell Auto Prep IFC, as specified by the manufacturer (protocol 100-7168 E1). The SMARTer Ultra Low RNA Kit (634936, Clontech) was used for cDNA synthesis from the single cells. Illumina NGS library was constructed with Nextera XT DNA Sample Prep kit (FC-131-1096, Illumina), according to the manufacturer's recommendations (protocol 100-7168 E1). Sequencing was performed on Illumina HiSeq2500 (Illumina) high output run mode by multiplexed paired end run with 60, 100 or 125 cycles.

The single cell RNA-seq reads were filtered with sickle for quality control following the parameter “sickle pe -q 30 -l 36 -g”, then trimmed using trim_galore as per parameter “trim_galore -q 25 --phred33 --length 50 -e 0.1 --stringency 3 --paired”. The trimmed sequences were aligned to the reference mm10 mouse genome with STAR-2.5.3a by using default parameters. Gene expression was quantified as raw counts using subread. To cluster the single cells into subgroups, PCA analysis with top 2000 highly variable genes was performed by using R package Seurat and projected on tSNE plots. Default parameters of the pipeline within Seurat were used to find the main clusters of single cells (FindClusters) with a customized setting for resolution = 0.7. Cluster marker genes were calculated using Wilcoxon rank-sum test contrasting each cluster against the rest and selected with the cutoff p value < 0.01. And, the following GO analysis of the marker genes was performed using DAVID 36. To get the CNVs information by using scRNA-seq data, R package inferCNV was used with the default parameters. And the scRNA-seq data of wild type mammary gland cells from our previous study was used as normal control 37. Pipeline M3Drop 39 was utilized for cluster and marker genes analysis of tumor cells within each tumor following the default parameter except the customized setting k = 3 for the number of sub-clusters. Top 20 marker genes for each sub-clustered were displayed in the heatmaps, while all the marker genes selected with AUC > 0.7 and p value < 0.05 were used for GO analysis via DAVID. The analysis on activation levels of hallmark pathways of cancer was performed by using GSVA based on the gene sets from MSigDB database (https://www.gsea-msigdb.org/gsea/msigdb/).

### Dropseq of single mammary cells and tumor cells

The single cell suspension dissociated from mouse mammary glands or mammary tumors (after epithelia enrichment) were loaded to the microfluidic droplet generator for dropseq as per the protocol described by McCarroll's lab 40. The dropseq libraries were constructed according to the same protocol and sequenced on Illumina HiSeq X Ten platform with paired-end 150 bp.

Dropseq datasets were processed with Dropseq tools v.1.13. Cell and molecular barcode tagging, primer and poly-A trimming, read alignment, barcode recovering, gene/exon annotations and barcode repairs were sequentially accomplished according to the online protocol (https://github.com/broadinstitute/Dropseq/files/2425535/DropseqAlignmentCookbookv1.2Jan2016.pdf). Read alignment was done using STAR aligner against Ensembl Mus musculus GRCm38.p6 assembly. Counts from all libraries were aggregated into MG1, MG2, MT1 and MT2 which represented mammary cells from mouse 1 and mouse 2, tumor cells from mouse 1 and mouse 2 respectively.

Cluster analysis was done with R package Seurat v. 3.0.2. For each count table, cells with less than 100 expressed genes and genes present in less than 4 cells were removed. The remaining counts were further filtered to remove 1% cells with excessively large or small numbers of genes, as well as 1% cells with excessively high percentages of mitochondrial genes. Cell cycle scores were calculated from 74 G1S genes and 43 G2M genes selected according to their GO annotations. Scaling and variable regression were then performed with SCTransform using negative binomial model to regress out the effects of percentage of mitochondrial genes and difference of cell cycle score from counts and then select the top 3000 highly variable genes for PCA. Truncated PCA was performed to estimate the top 30 principle components, of which the first N components were selected where N represented the turning point of elbow plot of standard deviations of principle components. The selected principle components were used to calculate the Euclidean distances of cells, which was then used to construct an SNN graph with k = 20, and clustering was done using standard modularity function, SLM algorithm for modularity optimization and resolution = 0.6. Clustering was visualized in t-SNE embeddings. Cluster marker genes were calculated using Wilcoxon rank-sum test contrasting each cluster against the rest. Constraints of a maximum of 500 cells in either group per contrast, only testing genes present in a minimum of 25% cells in either group and exhibiting log fold change higher than 0.25, and only including genes with positive fold changes, were applied. Genes with corrected p-value < 0.05 were retained as markers, adjusted by Bonferroni correction. A stricter marker identification was also performed with ROC method, with the same constraints except that genes with power > 0.8 were retained.

Combined analyses with more than one individual count table were performed by merging datasets from individual analyses. Filtering and cell cycle scoring were omitted, and the rest was done as previously described.

*Brca1*-deficient mammary tumorigenesis was reconstructed by trajectory analysis with R package monocle v. 2.10.1. As the mammary tumors were generated from the mammary epithelial cells, we removed the libraries of non-epithelial cells, including endothelial, immune cells and fibroblasts, before performing the monocle analysis. Combined count tables (filtered MG1+MT1, and filtered MG2+MT2) with cluster identities were imported from Seurat results. Lower detection limit was set as 0.5, and negative binomial size model was specified for size and dispersion estimations. The number of cells each gene was detectably expressed was calculated, genes which were expressed in less than 10% of cells were considered unexpressed. Differential expression analysis on expressed genes was done to identify genes differentially expressed between groups, genes with q-values < 0.01 were included as ordering genes for dimensionality reduction. Dimensionality reduction was done with DDRTree algorithm and maximum components = 2. Cells were ordered along pseudo-time trajectory and were assigned states according to their positions along the trajectory. Visual inspection and adjustment ensured mammary cells were located at the beginning. State markers, group markers and cluster markers were calculated using monocle differential gene testing routine with q < 0.01.

### CRISPR/Cas9 based gene knockout

For targeting murine *Mrc2* and other candidate genes, the relevant oligos (Table S1) were cloned into the lenti-CRISPR/Cas9-v2 vector (Addgene, #52961) following the Zhang lab protocol 41. The lentiviral plasmid, envelope plasmid (pMD2.G), and packaging plasmid (psPAX2) were co-transfected into 293FT cells with PEI to produce the lentiviruses. The culture medium was collected and filtered through a 0.45-µm filter at 72 h after transfection. The viral media were then 100× concentrated via PEG precipitation, resuspended with PBS, and harvested for infection. Target cells were infected with the enriched virus for 48 hours. Positive cells were selected for 5-7 days with puromycin (4 µg/mL and 8 µg/mL for B477 and G600 cells respectively) after infection.

For validation of target modification, genomic DNA was isolated from the single clones of targeted cell lines. Following PCR amplification of murine *Mrc2* (Forward: tcttcctcatcttcagccag, and Reverse: agtaaggtcgagcacatagg), the PCR products were sequenced. Allele modifications were determined by using the control cell as a reference sequence.

### Cell proliferation assay and drug sensitivity test

Cell proliferation was assessed by using alamarBlue cell viability reagent (DAL1100, Invitrogin) according to the manufacture's instruction. Briefly, 3000 cells were seeded into 96-well-plate containing 100 µl cell culture medium for 24-72 hours. The alamarBlue reagent was diluted as 1:10 with cell culture medium. Then the cells were incubated with the diluted alamarBlue reagent to replace the previous culture medium, for 3 hours in a cell culture incubator, protected from light. Then the fluorescence was measured and recorded by using a microplate fluorescence reader (SpectraMax® M5, Molecular Devices) with an excitation wavelength of 560 nm and an emission of 590 nm. All alamarBlue assays were performed at least 3 times in triplicate.

To test the sensitivity of mammary tumor cell lines to anti-cancer drugs, 3000 cells were seeded into 96-well-plate containing 100 µl cell culture medium. 24 hours later, the culture medium were changed to cisplatin (P4394, Sigma) or olaparib (AZD2281, Selleckchem) containing medium at given concentrations. Then the cell viability was test 72 after drug treatment by using alamarBlue cell viability reagent as mentioned above.

### Mammary fat pad allografts formation

Eight-week-old female nude mice used as recipients were obtained from the Animal Facility of the Faculty of Health Science, University of Macau. 2 x 10^5^ B477 or G600 cells suspended in 100 µl of 50% Matrigel (356235, Corning) plus 50% PBS were implanted into the fat pad of the nude mice. For each mouse, both flanks of the fourth pair of mammary fat pads were implanted. The mice were monitored for allografts growth every other day. And the allograft volume was calculated using the formula V = π/6xLxWxW, where L and W were the length and width of the allografts respectively.

### siRNA knockdown

siRNA oligos were designed by and purchased from Gene Pharma (Shanghai, China). The sequences of the siRNA oligos were listed in Table S2. The siRNA oligos were transfected into cells by using Lipofectine 2000 (11668019, Invitrogen) as per the manufacturer's instruction.

### Real-time PCR

Total RNA was extracted from the cells 48 after siRNAs transfection by using TRIzol reagent according to the manufacturer's instructions (15596026, Life Technologies). Reverse transcription to cDNA was initiated with 1µg of each RNA sample using a QuantiTect Reverse Transcription Kit (205313, Qiagen Inc.) following the manufacturer's instructions. Quantitative real-time PCR was performed using the Applied Biosystems™ QuantStudio™ 7 Flex Real-Time PCR System (ThermoFisher scientific). The reaction mixture contained 6 µl 2 × SYBR Green (04707516001, Roche), 4 µl PCR-qualified H_2_O, 0.5 µl forward primer (10 µM), 0.5 µl reverse primer (10 µM), and 1 µl of cDNA from the 100 µl diluted (1:5) stock solution prepared above. Each sample was analyzed in a 384-well PCR plate. The data were analyzed initially using SPSS 13.0 software (SPSS Company) included with the PCR machine. The results were analyzed statistically and graphed using Microsoft Excel 2010. The primers used for real-time reverse transcription PCR are shown in Table S1. *Actb* was used as an internal control.

### Statistics and reproductivity

Statistical analysis was performed with R software. An error bar represents mean ± s.e.m. Each experiment was repeated at least three times independently with similar results. Two-tailed Student's t-test was used to compare differences between groups. Differences in compared groups were considered statistically significantly different with *p* values lower than 0.05.

## Results

### Intertumor heterogeneity of BRCA1-defecient mammary tumors

Both human individuals with heredofamilial *BRCA1* mutations and BRCA1-deficient mouse models stochastically develop mammary tumors 8, 42-44, indicating multiple factors might contribute to the oncogenic process and lead to great heterogeneity of the tumors. To investigate the heterogeneity of BRCA1-deficient breast cancers, we firstly referred the human breast cancer cases from public data. Among 6113 breast cancer samples from different cohorts collected in cBioPortal, 169 (2.76%) harbored the genetic alterations on *BRCA1* (Figure S1A). As BRCA1 functions as a tumor suppressor, we only selected the cancer samples with loss-of-function mutations of BRCA1, including deep deletion, truncating mutation and missense mutation (putative driver) as BRCA1-deficient cancers for further analysis. Within 63 BRCA1-deficient cancers, related RNA-seq data were available from 13 samples in TCGA database (Figure S1 and Table S3). As these samples harbored somatic mutations of *BRCA1*, for comparison we included 16 TCGA breast cancer samples with germline* BRCA1* mutations, which were identified by Inagaki-Kawata et al. 29. Based on PAM50 calculation, these cancers are assessed as distinct molecular subtypes, with majority belonging to the basal-like subtype (Figure S1B and Table S3). We further checked expression levels of *ESR1*, *PGR* and *ERBB2*, and several marker genes for mammary basal (*KRT14*,* KRT17*) and luminal (*KRT8*, *KRT18*) lineages, epithelial (*CDH1*, *EPCAM*) and mesenchymal (*VIM*, *FN1*) cells. In line with the molecular subtyping result, different samples display distinct expression patterns of these genes, indicating great intertumor heterogeneity among both BRCA1 germline and somatic mutant breast cancers.

**Figure 1:**
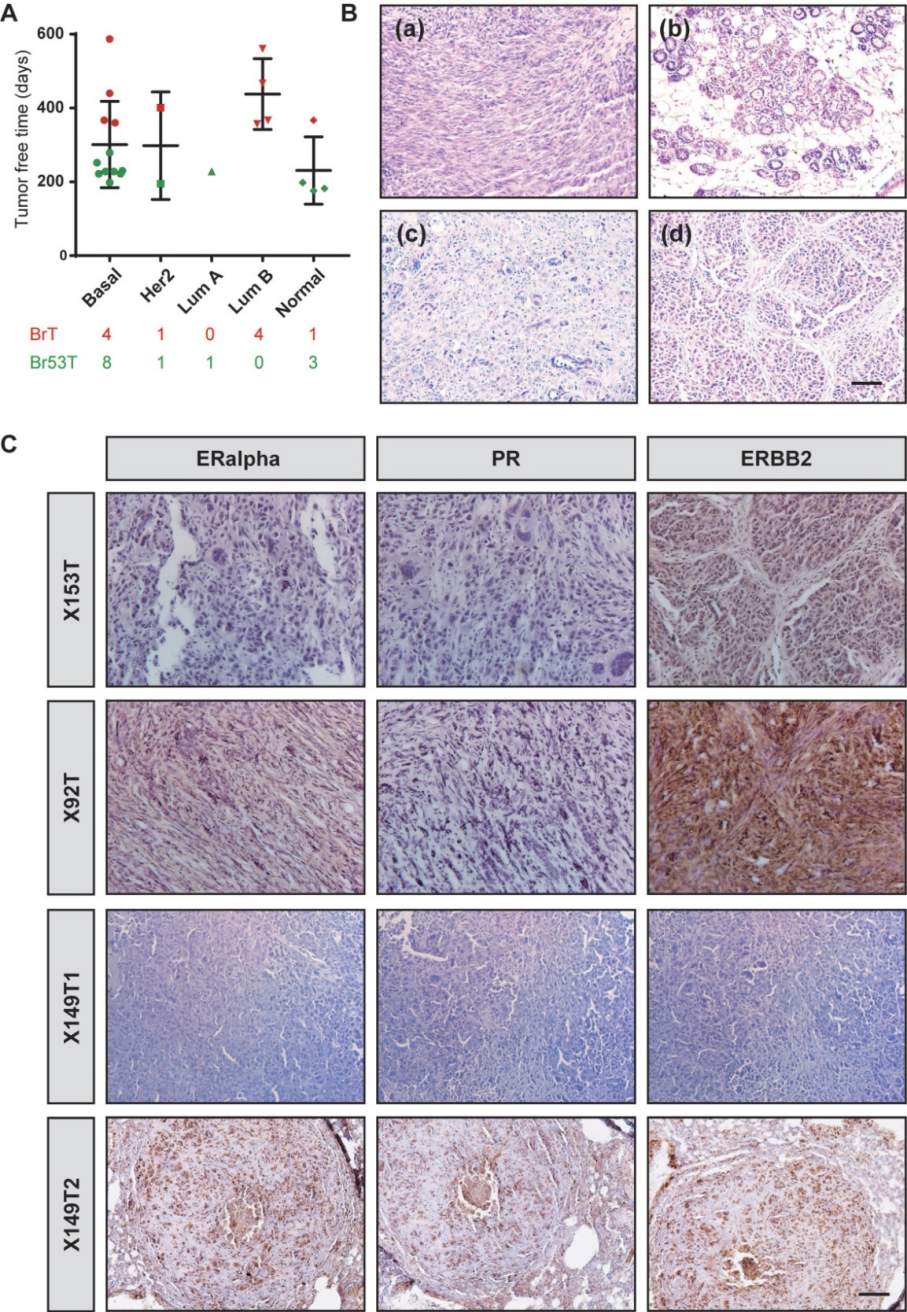
** Intertumor heterogeneity of BRCA1-defecient mammary tumors. A**. Summary of the tumor free time of 23 BRCA1-defecient mouse mammary tumors. The tumors are grouped as their PAM50 subtyping and marked according to their genotype. BrT (red), mammary tumors from *MMTV-Cre; Brca1^fl/fl^* mice; Br53T (green), mammary tumors from *MMTV-Cre; Brca1^fl/fl^; Trp53^fl/+^* mice. **B**. Distinct histopathological features of BRCA1-defecient mammary tumors are shown by H&E stained sections: (a), one tumor with mesenchymal/fibroblast-like feature; (b), one tumor with glandular structure; (c), one tumor with medullary feature; (d), one tumor with net-like feature and typical pushing margins. **C**. Distinct expression patterns of ERalpha, PR and ERBB2 in BRCA1-defecient mammary tumors are shown by IHC stained sections. Scale bar, 50 µm.

To further study the heterogeneity of BRCA1-deficient mammary tumors, we collected 23 tumors from two previously established mouse models, where *MMTV-Cre* driven loss of BRCA1 (*MMTV-Cre*;* Brca1^fl/fl^*) specifically develops mammary tumors (BrTs) in 1-1.5 year, while combination of *Trp53* mutation (*MMTV-Cre*;* Brca1^fl/fl^*;*Trp53^fl/+^*) could greatly accelerate the tumorigenesis (Figure 1A). Among the tumors, 10 were BrTs and the other 13 were collected from double mutant mice (termed as Br53Ts). Molecular subtyping of the tumors demonstrated that majority of the tumors are basal-like, while other subtypes also exist (Figure 1A), which is similar to human BRCA1-deficient cancers (Figure S1). Besides, we examined the histological features of these tumors, and found overt intertumor diversity. Some tumors are characterized with mesenchymal/fibroblast-like feature, while others with glandular structure, medullary feature or net-like feature (Figure 1B). Majority of human BRCA1-related breast cancers are negative for ERα, PR and/ or ERBB2. So, we checked the expression levels of these receptors in histological sections of mouse tumors (Figure 1C). About 78.2% of the BRCA1-deficient mammary tumors do not express ERα or PR, though about 60% of the tumors showed detectable level of ERBB2 (Figure S2A, Table S4). Taken together, these results manifested that the BRCA1-deficient mammary tumors bear great intertumor heterogeneity.

### Molecular subtyping of BRCA1-defecient mammary tumors

**Figure 2:**
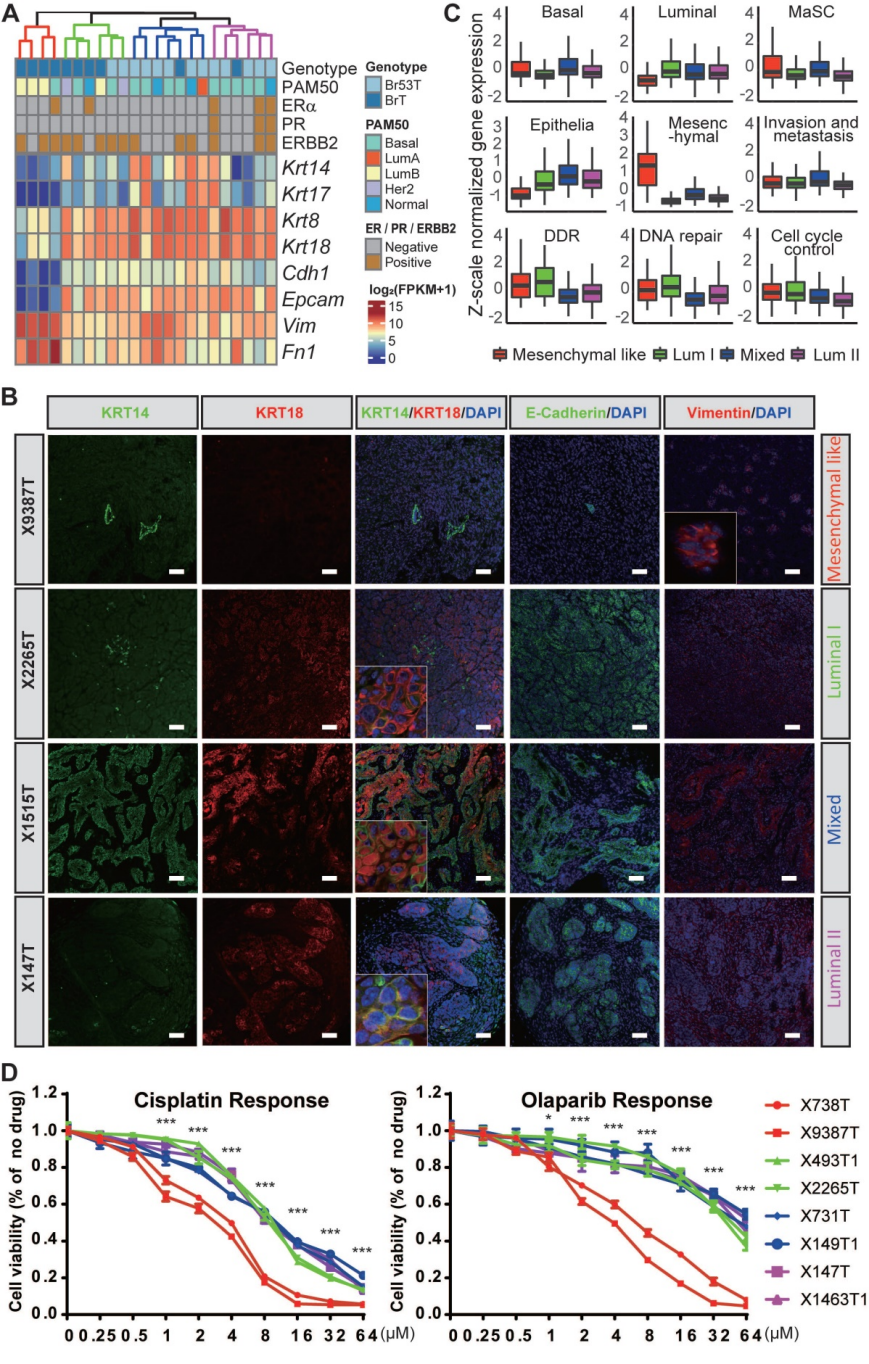
** Molecular subtyping of BRCA1-defecient mammary tumors. A**. 23 BRCA1-defecient mammary tumors are hierarchically clustered into four subgroups based on whole transcriptome. The genotype, intrinsic cancer subtype based on PAM50, ERα/PR/ERBB2 expression patterns based on IHC staining as well as mRNA levels of several selected genes are shown. **B**. The expression patterns of KRT14, KRT18, E-Cadherin and Vimentin in 4 BRCA1-defecient mammary tumors are shown by immune-fluorescence stained sections. The 4 tumors were from 4 distinct subgroups shown in panel **A**. Scale bar, 50µm.** C**. Boxplots representing Z-scale normalized gene expression values from 4 subgroups of BRCA1-defecient mammary tumors show expression levels of different groups of genes (Figure S3). The box represents the interquartile range and the line is the median. **D**. The sensitivities of 8 *Brca1*-mutant mouse mammary cell lines to cisplatin (left panel) and olaparib (right panel). The colors of the lines represent the subtypes of the mammary tumors where the cell lines were derived. Wilcox-rank sum test was conducted for the analysis of drug sensitivity between mesenchymal-like tumors and the other 3 groups. ns, no significance; *, P < 0.05; **, P < 0.01, and ***, P < 0.001.

Next, we wanted to classify the heterogeneous mammary tumors with their distinct molecular characteristics. Based on the whole transcriptomes of the 23 BRCA1-deficient mammary tumors, four subgroups are identified via hierarchal clustering, and termed as mesenchymal like, luminal like I and II (Lum I and Lum II), and mixed types respectively because of their differential expression patterns of mammary basal/luminal lineage markers and epithelial/mesenchymal cell markers (Figure 2A-B). For example, the mesenchymal like tumors highly express mesenchymal markers *Vim* and *Fn1*, compared with other tumors. The tumors of luminal like subgroups highly express luminal markers *Krt8* and *Krt18*, while displaying low expression levels of basal markers *Krt14* and *krt17*. Moreover, the mixed type tumors express both the basal and luminal markers. We also carefully examined the histopathological features of the mammary tumors, and found the mesenchymal like tumors display low differentiation level and typical fibroblast like morphology, while the luminal like tumors are in relatively high differentiation level (Figure S2B).

To further dissect the molecular features of the mammary tumors, we checked the relative activation levels of several key pathways involved in mammary development and tumorigenesis, and/or biological functions of BRCA1 (Figure 2C and Figure S3A). Notably, the mammary stem cell (MaSC) markers are highly expressed in the mesenchymal like and mixed type tumors, and the activity of invasion and metastasis is also higher in these tumors than the luminal like ones. Additionally, the activity of invasion and metastasis is even higher in the mixed type tumors compared with the mesenchymal like ones, indicating the tumor cells in the intermediate state of EMT possess better capacity of invasion and migration than the cells in the complete state of EMT, which is consistent with previous reports 45, 46. We also identified marker genes for each subgroup of the mammary tumors and summarized the enriched biological functions of the markers via gene ontology (GO) analysis using DAVID 36 (Figure S3B, and Table S4).

Further, we wondered whether different subtypes of BRCA1 deficient mammary tumors bear heterogeneous sensitivity to anti-cancer drugs. To test this, we established several mouse mammary tumor cell lines and determined their sensitivities to cisplatin and olaparib. And notably, the mesenchymal-like mammary tumor derived cell lines are more sensitive to both drugs (Figure 2D), indicating these tumors may be more sensitive to the DNA damage inducers and PARP inhibitors. We further checked the activities of anticancer drug resistance related pathways, including DNA damage response (DDR), DNA repair, ATP-binding cassette (ABC) efflux, shieldin complex and cell death regulation, based on the expression levels of relevant gene sets among tumors of different subtypes. The results indicated none of these pathways were associated with the high sensitivity of mesenchymal like tumors to cisplatin and olaparib (Figure S4). In addition, previous study reported that the decelerated proliferation and cell dormancy contribute to the multidrug resistance of tumor cells 47. Indeed, we found the mesenchymal like tumor cells grow much faster than other subtypes (Figure S4G), which might result in their high sensitivity to the drugs.

Taken together, the BRCA1 deficient mammary tumors could be classified into four distinct subtypes, with respective markers genes, molecular features and anti-cancer drug sensitivities.

### Single cell analysis of BRCA1-defecient mammary tumor cells and luminal cells

**Figure 3:**
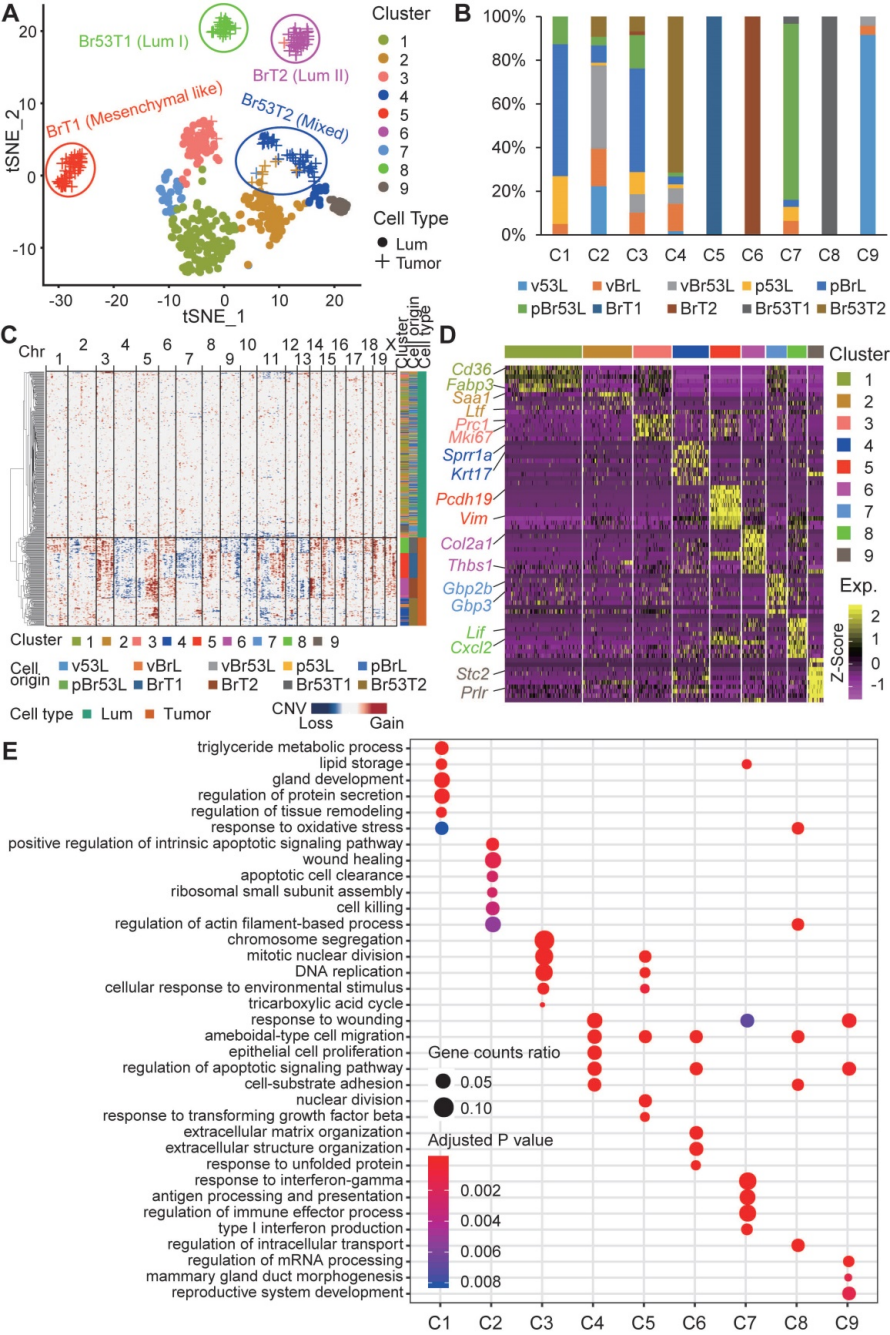
**Single cell RNA sequencing of sorted BRCA1-defecient mammary luminal and tumor cells. A**. tSNE plot displays the clustering of single BRCA1-defecient mammary luminal cells and tumor cells. The cells are clustered into 9 groups based on Seurat analysis and respectively labeled accordingly. **B**. Summary of the cells composition for each cluster. **C**. Heatmap shows the normalized CNV levels of single luminal and tumor cells. The red and blue colors represent copy number gain and loss, respectively. **D**. Heatmap shows the expression levels of marker genes for each cluster (Table S6). Some representative markers are highlighted on the left. **E**. Bubble diagram shows the representatives of enriched GO terms for each cluster.

The observation of great intertumor heterogeneity among BRCA1-deficient mammary tumors propelled us to posit whether such phenomenon is ascribed to the intratumor heterogeneity among tumor cells. To address this, we resorted to the Fluidigm C1 platform based scRNA-seq to decipher the composition of BRCA1-deficient mammary tumor cells, as well as the mammary luminal cells, in which loss of BRCA1 initiates the tumorigenesis (Figure S5, and Table S5). Totally, we performed scRNA-seq on 165 tumor cells from four mammary tumors of distinct molecular subtypes (termed as BrT1, BrT2, Br53T1, and Br53T2; Table S5), as well as 312 luminal cells from female mice with three different genotypes (*Brca1* mutant; *Trp53* mutant; and *Brca1/ Trp53* double mutant, Table S5). To study whether pregnancy affects the gene expression patterns of mammary cells with different genotypes, we selected luminal cells from both 3-month-old virgin mice and age-matched pregnant mice at day 12.5 (P12). We firstly applied the principle component analysis (PCA) on all the mammary luminal cells and tumor cells with the Seurat R package 48, and identified 9 main clusters (Figure 3A). When projected into the t-distributed Stochastic Neighbor Embedding (t-SNE) plot, we found that the luminal cells with different genotypes are roughly clustered together, indicating there is little inter-individual heterogeneity (Figure 3A). However, the tumor cells appear overt intertumor heterogeneity (Figure 3A-B). Meanwhile, regardless of genotypes, the luminal cells from virgin mice are mostly grouped in C2 and C9, while the luminal cells from P12 mice are mainly grouped in C1, C3 and C7, which indicates the pregnancy vastly influences the gene expression profiles of luminal cells and overrides the effect of loss of BRCA1 and/or TRP53.

We also calculated chromosomal copy number variations (CNVs) in each cell using InferCNV 49 (Figure 3C). And a great many of CNVs are observed across almost all the chromosomes in the tumor cells. Besides, we noticed a few luminal cells also harbor some CNVs, especially on chromosomes 10, 11 and 12. These cells are perhaps at an early stage of oncogenic transformation.

To further elucidate the molecular variations among the single cells, we checked the marker genes for each cluster (Figure 3D, and Table S6). We found that lipid and fat metabolism related genes like *Cd36* and *Fabp3* are highly expressed in C1, and also moderately expressed in C3 and C7, which is in accordance with the pregnant situation of donor mice. Almost all the BrT1 cells (C5) highly express the mesenchymal marker *Vim*, which is in line with the bulk RNA-seq result (Figure 2A). Interestingly, *Prlr* is specifically expressed in C9 cells, most of which are luminal cells from virgin *Trp53* mutant mice, indicating both the pregnancy (P12) and deficiency of BRCA1 may block the expression of *Prlr.* Next, we examined the enriched biological functions of the cluster-specific marker genes (Figure 3E). Notably, tumor cell related clusters (C4, C5, C6 and C8) all enrich the GO term of ameboidal-type cell migration, indicating it might be a common event that tumor cells gain the capacity of ameboidal migration after oncogenic transformation.

To acquire more details of differences among the mammary luminal cells, we performed the clustering analysis only on the luminal cells (Figure S6A). These cells are divided into 5 subgroups (Lum1-5). While subgroups Lum1, Lum3 and Lum5 are mainly composed of luminal cells from P12 mice, luminal cell from virgin mice are mostly classified into Lum2 and Lum4. Every subgroup includes cells with three distinct genotypes, only except Lum5, which is dominated by luminal cells from P12 *Brca1*/*Trp53* double mutant mice. Marker genes and related GO analysis of each subgroup reveal that genes involved in immune system process like *Cxcl9*, *Cxcl10*, *Ifi47* are highly expressed in Lum5 cells (Figure S6B-C). This indicates loss of BRCA1 and TRP53 might induce immune response in some luminal cells.

Then, we performed the similar analysis on all the mammary tumor cells (Figure S6D-F). And we noticed that the BrT1 cells are divided into two groups, Tum4 and Tum5. Though both groups share some marker genes, Tum5 cells are in a more proliferative state, which is indicated by the induced expression of cell cycle related genes *Top2a*, *Cdk1* and so like (Figure S6D-F). Besides, we found Tum1 and Tum2 are clustered closely on the tSNE plot (Figure S6D). While all the Br53T1 cells together with a few Br53T2 cells are clustered as Tum2, the rest Br53T2 cells are classified into Tum1, which indicates there might be some similarity of transcription regulation among *Brca1*/*Trp53* double mutant mammary tumors. And GO analysis result of marker genes for both clusters manifests that biological function like response to virus and immune system process is significantly enriched in both clusters (Figure S6F), which has also been identified in part of double mutant luminal cells (Figure S6B-C).

### Intratumor heterogeneity of BRCA1-defecient mammary tumors

**Figure 4:**
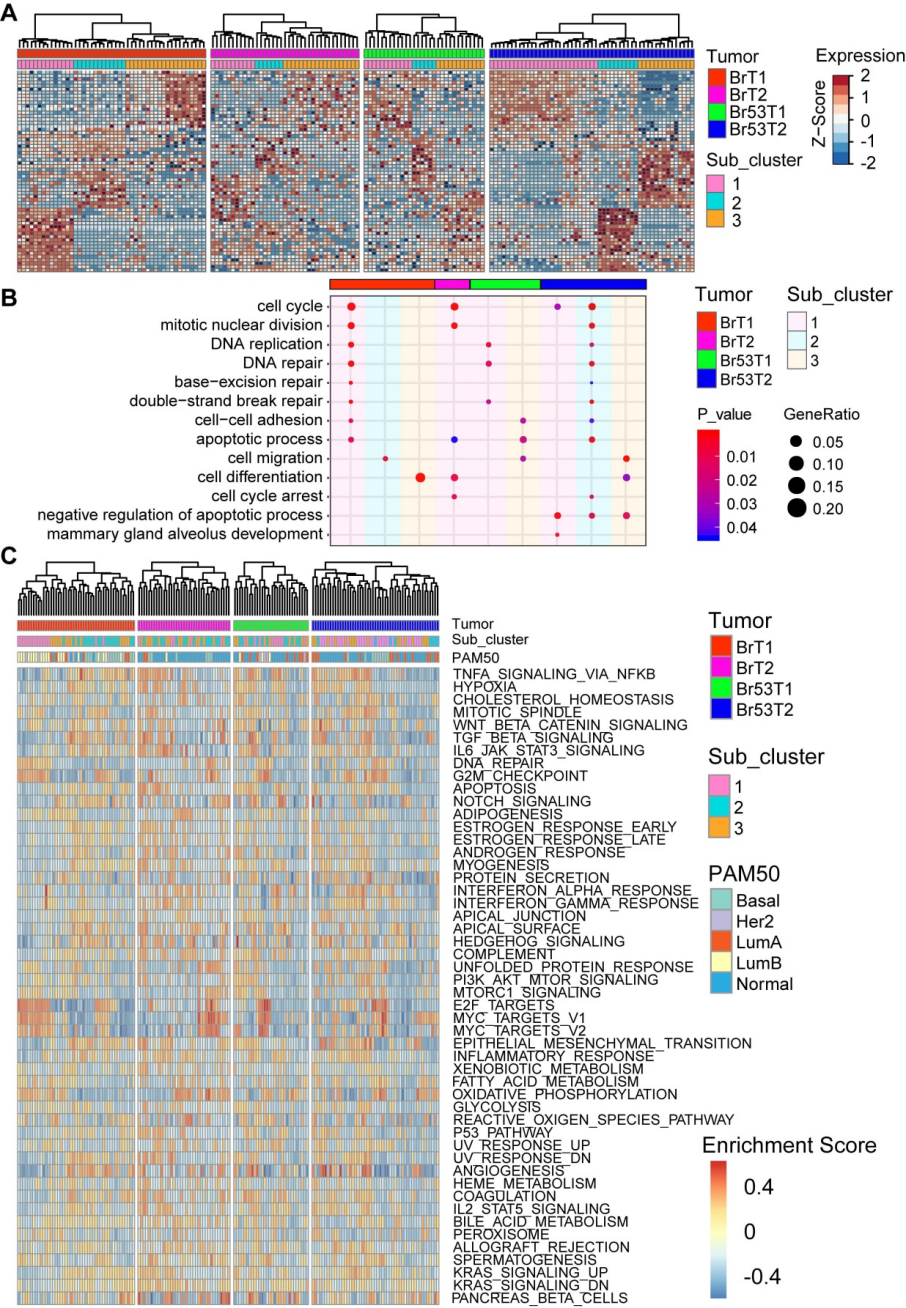
** Intratumor heterogeneity of BRCA1-defecient mammary tumors revealed by single cell RNA sequencing. A**. Heatmap shows the expression patterns of marker genes for each sub-cluster within individual mammary tumor. Single cells of each tumor are divided into three sub-clusters based on M3Drop analysis (Table S7). **B**. Bubble diagram shows the representatives of enriched GO terms for each sub-cluster of individual tumor. **C**. Heatmap shows the heterogeneous activation of biological hallmarks pathways among single mammary tumor cells. The intrinsic cancer subtype of the single tumor cells based on PAM50 scaling is shown (above) as well.

As the intertumor heterogeneity was much higher than the intratumor one, we next hierarchally clustered the single tumor cells separately according to their tumor identity via another R program M3drop 39. As shown in Figure 4A, the single cells within each tumor could be further divided into three subgroups, with individual marker genes, which enrich distinct biological functions (Figure S7, and Table S7). Notably, the GO terms enriched by the marker genes for subgroups demonstrate that the main diversity among three subgroups within individual tumor are concerned with cell cycle control, DNA repair, cell migration, apoptosis, and mammary gland development (Figure 4B, and Figure S7). Next, in each individual tumor cell, we checked the molecular subtyping via calculation of PAM50 (Figure 4C). In line with previous studies 50, 51, the result showed that various subtypes of breast cancer could be identified on the single cell level, though the majority are normal-like, which accounts for about 46% (76/165) of all the tumor cells (Table S5). In addition, we evaluated the activation levels of biological hallmark pathways via GSVA analysis 52 (Figure 4C). Most of the pathways are activated in part of the single tumor cells with different levels. Especially, cell proliferation related pathways like E2F_targets and MYC_targets are highly activated in some of the tumor cells within all four tumors. And immune response related pathways such as TNFα_signaling via NFKB, TGFβ_signaling, IL6_JAK_STATs_signaling also display high activity, especially in the cells with low proliferation (Figure 4C). In sum, there is great intratumor heterogeneity among single cells based on analysis of the gene expression patterns, PAM50 subtyping and the activity of biological hallmark pathways.

### Dropseq to reveal molecular changes during BRCA1-deficient mammary tumorigenesis

**Figure 5:**
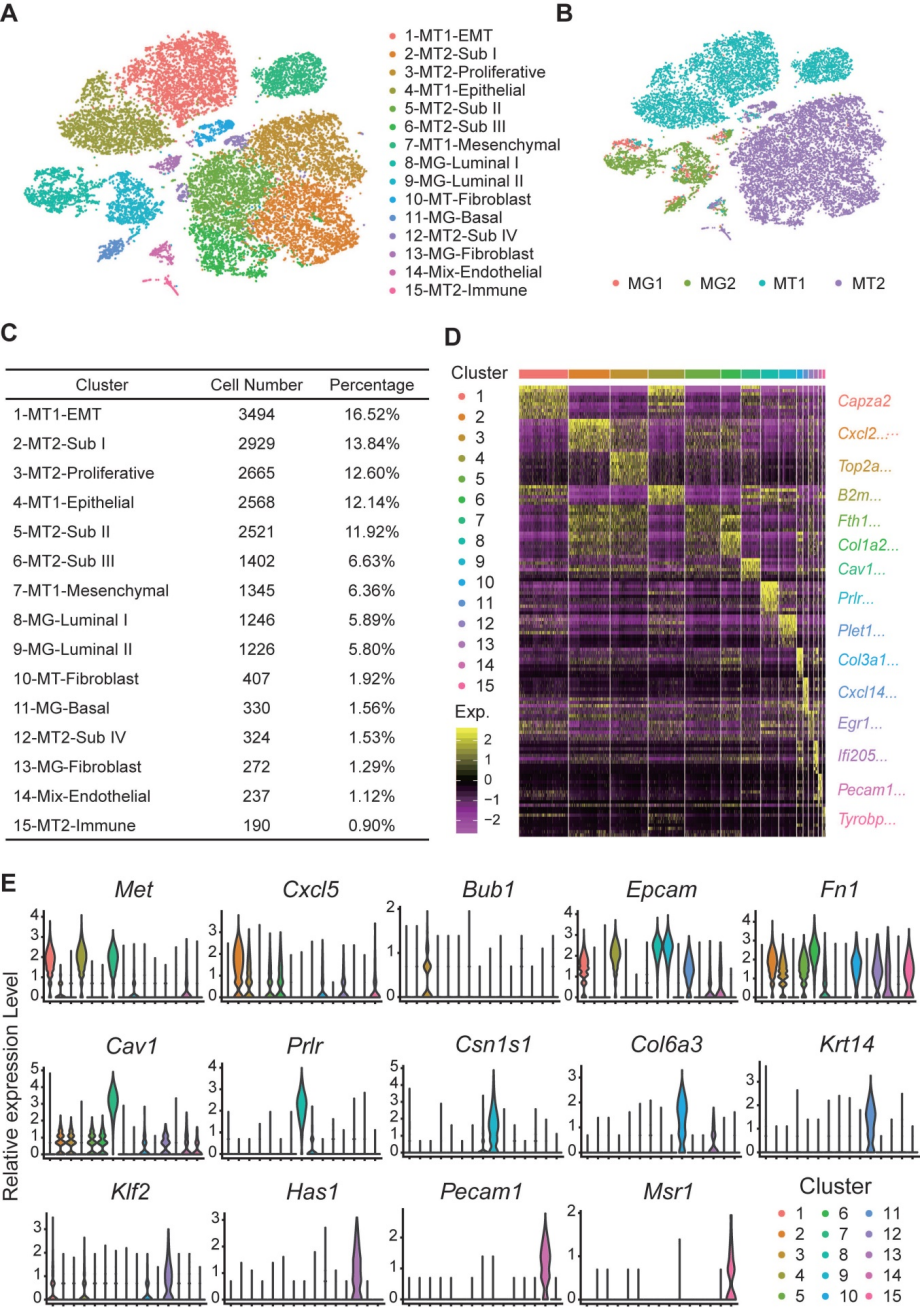
**Dropseq of paired mammary gland (MG) cells and mammary tumor (MT) cells from 2 *Brca1* mutant mice. A** and **B**. tSNE plots demonstrate the cell types and groups of the MG and MT cells (Table S8). The cells are divided into 15 groups, which are separately clustered and respectively labeled with different colors in panel **A**. The cells origin is shown in panel **B**. **C**. Summary of cell number and percentage of each group of cells. **D**. Heatmap displays the expression patterns of marker genes for each group of cells (Table S9). **E**. Violin plots show the expression patterns of representative markers across the cell groups. The y-axis indicates the normalized read count.

After analysis of inter- and intra-tumor heterogeneity of the BRCA1-deficient mammary tumors, we wanted to further explore how the mutant mammary cells transform into tumor cells. Here, we applied dropseq to analyze pair-matched mammary gland cells and mammary tumor cells from two *Brca1* mutant mice (termed as MT1 and MT2 hereafter). Totally, 21157 cells including 3153 mammary cells and 18004 mammary tumor cells with high-quality sequencing data were analyzed and classified into 15 main clusters (Figure 5A). Then, we identified the cell type of each cluster according to the cell origin and marker genes (Figure 5A-C, and Table S8). The representative marker genes of each cluster are shown in Figure 5D-E (Table S9).

**Figure 6:**
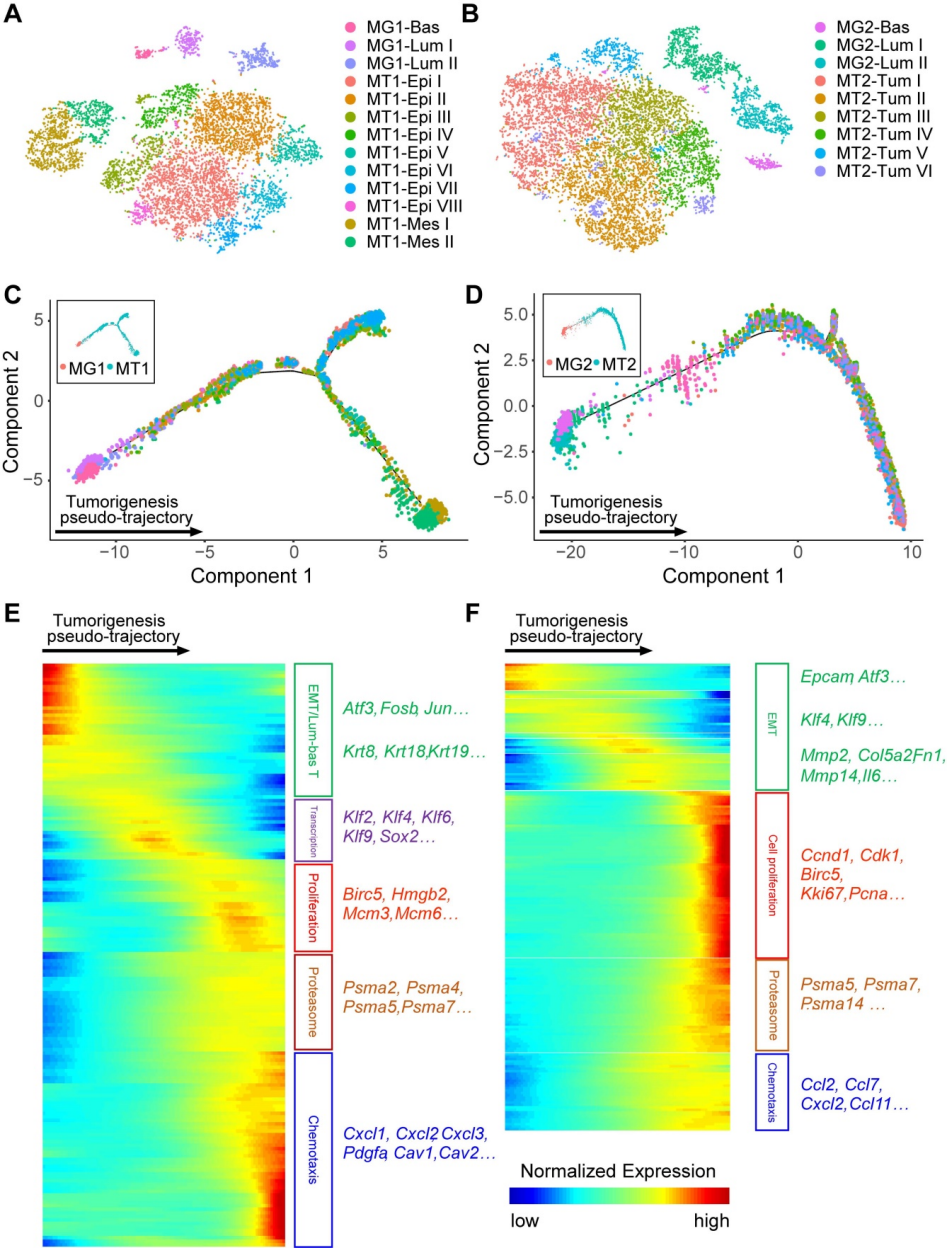
** Molecular changes during BRCA1-deficency induced tumorigenesis. A** and **B**. tSNE plots demonstrate the cell types and groups of the filtered MG and MT cells from two individual mice. The single MG and MT cells from mouse 1 and 2 are divided into 13 (**A**) and 9 sub-groups (**B**) respectively. **C** and **D**. Monocle analysis reveals the pseudo-temporal trajectories of tumorigenesis in mouse 1 (**C**) and 2 (**D**). **E** and** F**. Heatmaps show the expression patterns of differentially expressed genes (rows) along the pseudo-time (columns) of tumorigenesis (left panels, Table S10). The enriched biological functions and representative markers for gene clusters with similar expression pattern are shown in the right panels.

To uncover the molecular changes during BRCA1-deficency induced tumorigenesis, we separated the single cells (only mammary epithelial and tumor cells) as per their mouse origin because the tumorigenesis process may be different among individuals. Among the single cells from mouse 1, 13 subgroups including 1 MG basal, 2 MG luminal, 8 epithelial MT and 2 mesenchymal MT clusters are identified (Figure 6A). Similarly, 1 MG basal, 2 MG luminal, and 6 MT subgroups are identified in mouse 2 (Figure 6B). Then, we performed the monocle analysis to reconstruct the pseudo-temporal trajectories of the tumorigenesis 53 (Figure 6C-D). And three continuous states along the pseudo-temporal trajectories are classified (Figure S8A-B, and Table S10). Next, we further examined the differentially expressed genes (DEGs) with distinct variation tendencies along the pseudo-temporal trajectories (Figure 6E-F). Notably, we found a consistent EMT process during tumorigenesis in two mice, with expression of epithelial markers like *Epcam* and *Atf3* are gradually reduced. And at the end stage of tumorigenesis, genes related to cell proliferation, proteasome and chemotaxis are highly expressed in tumor cells of both mice.

Besides, we further checked some important signaling pathways related to mammary development and tumor initiation and progression, to determine whether their activities are changed during the tumorigenesis process (Figure S8C). We noticed that expression of the epithelial markers was instantly shut down at the mammary cells-tumor cells joint along the pseudo-trajectory, which might be the oncogenic transformation turning point, while the mesenchymal markers are gently induced across this turning point (Figure S8C). Interestingly, expression of markers for basal cells and MaSCs shows a down-and-up tendency, with the lowest activity at the transformation turning point. Meanwhile, expression of markers for luminal cells is greatly reduced during the tumor initiation stage. All the results indicated the mammary cells would shut down the lineage specific genes and loss their transcription control of intrinsic features once the tumorigenesis initiates. However, the expression of basal and MaSCs marker genes may be required for the survival and/or expansion of the tumor cells, and so gradually induced in the late stages.

In addition, signaling pathways regarding cell cycle control, DNA damage response, survival-cell death regulation, invasion and migration, PI3K-AKT, Notch, and MAPK signaling all appear the similar down-and-up variation tendency (Figure S8C), indicating the overall transcription activity might be reduced to a very low level at the oncogenic transformation turning point. Taken together, our analysis of dropseq data provides a panorama of the molecular changes during BRCA1 deficiency induced mammary tumorigenesis process.

### Identification of DEGs between BRCA1-deficient luminal and tumor cells

In our mouse models, the *Brca1* mutation in luminal cells results in mammary tumors formation 10, 54. So, we wondered whether any of the DEGs between *Brca1* mutant luminal and tumor cells could serve as driver and/or marker for the tumorigenesis. Therefore, we compared the whole transcriptomes of luminal cells and tumors in single cell and bulk RNA sequencing data (Figure S9A-B, and Table S11). We found more than half of the DEGs discovered in bulk RNA-seq are overlapped with the DEGs identified in scRNA-seq (Figure S9C). Next we checked the biological functions of these common DEGs via GO analysis, and found fatty acid and lipid metabolism related pathways are significantly enriched in the luminal cells while cell adhesion, migration and immune response *etc*. are activated in the tumor cells (Figure S9D). Then, we ranked the DEGs by the fold-change of expression levels, and compared the top 100 DEGs for luminal and tumor cells separately, in both single cell and bulk RNA-seq data. We found 41 and 25 common genes are highly expressed in luminal cells and tumor cells, respectively (Figure S9E-G). Notably, 5 collagen-encoding genes show up as tumor DEGs. We just picked two of them as well as other 15 tumor DEGs for further functional validation. Meanwhile we chose 10 luminal DEGs for the parallel validation. Some tumor DEGs were excluded for further investigation because their functions in mammary tumorigenesis have been well studied, such as *H19* 55, *S100a4* 56, 57, *Rspo1* 58, 59*,* etc.

**Figure 7:**
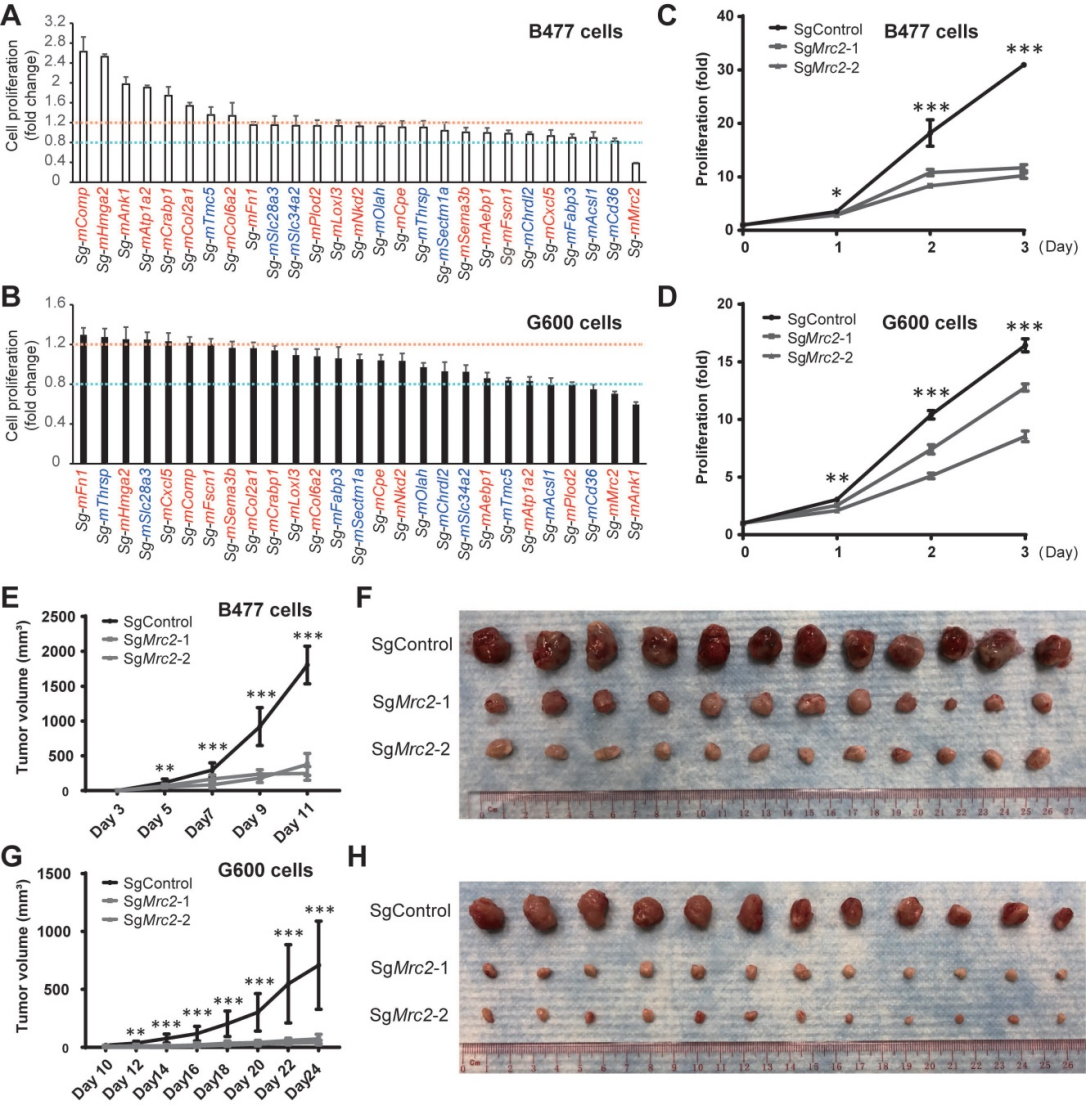
***Mrc2* serves as an oncogene in mammary tumorigenesis. A** and **B**. Cell proliferation screening of candidate driver genes identifies a pro-proliferation role of MRC2 in mammary tumor cells. 27 candidate gens were selected and knocked out individually in B477 cells (**A**) and G600 cells (**B**). The genes with red color or blue color indicate their up-regulation or down-regulation in the mammary tumor cells compared with luminal cells (Table S11). The cell proliferation was measured with alamarBlue assay 72 hours after cells were seeded. The cells with non-target sgRNA were used as control for cell proliferation fold change calculation. The fold change > 1.2 or < 0.8 was used as cutoff for cell proliferation induction (orange line) or reduction (cyan line), respectively. **C** and **D**. Cell growth curves indicate the proliferation was significantly inhibited after *Mrc2* knockout. Two different sgRNAs were used to target *Mrc2* in B477 cells (**C**) and G600 cells (**D**). **E-H**. Knockout of *Mrc2* blocked tumor cells growth *in vivo*. The tumor growth of implanted *Mrc2* knockout B477 (**E** and** F**) or G600 (**G** and** H**) cells was significantly blocked in nude mice. The tumor volume changes (**E** and** G**) and photos of allografts (**F** and** H**) are shown. *, p value < 0.05; **, p value < 0.01; ***, p value < 0.001.

Accelerated proliferation is a very significant and general hallmark of tumor cells. To test whether the candidate genes are involved in the regulation of cell proliferation, we knocked out them separately via CRISPR/Cas9 system in both *Brca1* wild type *Trp53* mutant B477 mammary epithelial cells and *Brca1* mutant G600 mammary epithelial cells 20. Compared with the non-targeting control cells, proliferation level is altered after knockout of several genes, among which loss of *Mrc2* could reduce the cell growth in both lines (Figure 7A-B).

### *Mrc2* serves as an oncogene in mammary tumorigenesis

As knockout of *Mrc2* inhibits cell growth in both B477 and G600 cells, we wanted to further study the function of MRC2 in mammary tumors. Previous study reported that MRC2 is mainly expressed in the stromal cells but not the epithelial cells of mammary glands 60 and only expressed in some breast tumor cells 61. By carefully checking the expression patterns of MRC2 in histological sections of *Brca1* mutant mouse mammary glands and tumors, we found MRC2 is expressed at low level in the basal cells and highly expressed in the tumor cells (Figure S10A). Bulk RNA-seq data confirmed such expression patterns (Figure S10B). We further checked the expression level of *Mrc2* in the 4 subtypes of BRCA1-deficient mouse mammary tumors, and found *Mrc2* was highly expressed in the mesenchymal-like tumors when compared to other 3 subtypes (Figure S10C). We also found the expression of MRC2 could be detected in some normal and cancer tissues of human breast samples collected in public database, the human protein atlas (Figure S10D). And the overall protein level of MRC2 is significantly higher in the breast cancers compared with normal tissues among the human samples collected in the Clinical Proteomic Tumor Analysis Consortium (CPTAC) dataset 32 (Figure S10E).

In addition, consistent with the basal-like feature of *Brca1* mutant cancers, G600 cells adapt morphology more like mesenchymal feature compared with *Brca1* wild type B477 cells, which display epithelial morphology (Figure S11B). Notably, sg*Mrc2*-G600 cells undergo a morphological conversion from mesenchymal to epithelial transition (MET), as the sg*Mrc2*-G600 cells appear epithelial feature and get attached to each other (Figure S11B). In contrast, sg*Mrc2*-B477 cells exhibit very little morphological change (Figure S11B), despite reduced proliferation has been observed. Moreover, knockout of *Mrc2* with another targeting sgRNA also reduces the proliferation of B477 and G600 cells (Figure 7C-D). And knockdown of *Mrc2* with two different siRNAs confirmed the essential role of *Mrc2* in proliferation of G600 cells (Figure S12A-B). Next, we examined whether knockout of *Mrc2* could also block the tumor cell growth *in vivo* by implanting the cells into the fat pads of nude mice. The results demonstrated that tumor formation capacity of both B477 and G600 cells is obviously inhibited in the knockout groups compared with the control cells, especially in the *Brca1* mutant G600 cells (Figure 7E-H, and Figure S12C and D). In addition, we found knockdown of *Mrc2* in cell lines derived from *Brca1* mutant tumors significantly blocked cell growth and reduced the mRNA levels of proliferation markers *Ccnd1* and *Mki67* in regardless of their subtypes (Figure S12E-L). Besides, knockdown of *MRC2* in human breast cancer cell lines: MCF7, MDA-MB-231, and MDA-MB-436 could also decelerate the cell proliferation, and reduce the mRNA levels of proliferation markers *CCND1* and *MKI67* (Figure S12M-R). Taken together, *Mrc2* may serve as an oncogene in the mammary tumorigenesis due to its essential role in controlling cell proliferation.

## Discussion

In this study, we explored the inter- and intra-tumor heterogeneity of BRCA1 deficient mouse mammary tumors. On the intertumor level, we uncovered great heterogeneity of histopathological features, expression patterns of ER/PR/ERBB2, molecular subtyping, and transcriptome profiles among individual tumors, and identified 4 subgroups with distinct molecular characteristics and marker genes, and also different sensitivities to anti-tumor drugs. On the intratumor level, we revealed the main gene expression variations and the differences of the enriched biological functions among subgroups of cells within individual tumors.

As the first identified breast cancer associated gene, *BRCA1* is closely associated with mammary gland development and tumorigenesis. Although the mutation frequency of *BRCA1* is not high among breast cancer patients, women with germline mutation of *BRCA1* would reach a breast cancer risk as high as 50%-80% during their lifetime 62. More importantly, *BRCA1* mutations are always linked to the refractory type of breast cancers, termly the basal like breast cancers, always also TNBCs 2. In this study, we found near 80% of the BRCA1-deficient mouse mammary tumors are ER and PR double negative, while 60% of the tumors show detectable expression level of ERBB2 (Figure S2A), which is inconsistent with the human BRCA1 related breast cancers 16. One BRCA1 associated genetic alteration 17q23 gain might lead to the amplification of *Erbb2 63*. As the 17q23 gain identified in BRCA1 related human breast cancers, which is far away from the *ERBB2* and *BRCA1* loci on human chromosome 17, is quite near the* Erbb2* and *Brca1* loci on mouse chromosome 11 64. More studies are required to pinpoint whether the expression of ERBB2 plays a driver or passenger role in the BRCA1-deficient mouse mammary tumors.

Another genetic alteration frequently occurred in *BRCA1* related breast cancer is the *TP53* loss, which is also observed in the BRCA1-deficient mouse mammary tumors 10. Besides, synchronous one allele loss of *Trp53* not only greatly accelerates the BRCA1 deficiency induced mammary tumorigenesis (Figure 1A), but also affects the molecular features of the tumors. We noticed that all the mesenchymal like mammary tumors in our cohort were generated from the *MMTV-Cre*;* Brca1^fl/fl^*;*Trp53^+/+^* mice (Figure 2A), which is contradictory to previous reported role of TRP53 in EMT, where loss of TRP53 could activate EMT through regulation of microRNAs-ZEB1/2, and MEK-ERK signaling, as well as other mechanisms 65-67. In consideration of the high mutation frequency of *Trp53* in BRCA1 deficient mammary tumors, and the much longer period for formation of Br53Ts than BrTs (Figure 1A), the mesenchymal like BrTs may generate and accumulate *Trp53* mutation during the tumorigenesis process. This raises the hypothesis that the timing of TRP53 loss may determine the subtype of BRCA1-deficient mouse mammary tumors. Actually, timing of TRP53 loss has been reported to affect the subtype of mammary tumors induced by* Rb* deficiency and *Pten* deficiency 68, 69. In addition, synergetic loss of BRCA1 and TRP53 may greatly induce the immune response of the mammary luminal cells and tumor cells, as compared with the *Brca1* mutant cells, the *Brca1/Trp53* double mutant cells, no matter luminal or tumor cells, display higher activity of immune response related pathways (Figure S6). Hence, more studies are still needed to figure out the functions and mechanisms of TRP53 on BRCA1 deficiency induced mammary tumorigenesis.

The BRCA1 deficiency induced mammary tumorigenesis is a continuous and dynamic process. During this process, lots of stochastic mutations and variable levels of DNA damage induced stress could be accumulated in individual cells, which might greatly affect the gene expression patterns among the cells, and lead to the intratumor heterogeneity. Meanwhile, the intratumor heterogeneity could also result from the intrinsic cell cycle differences (different cells at different cell cycle stages) and mammary hierarchical variations (different cells at different differentiation stages and mammary lineages). The above heterogeneous features could be also observed on the intertumor level. In addition, a few recently published studies provided more information about the inter- and intra-heterogeneity of BRCA1-deficient mammary tumors/breast cancers. For example, Wang, et al. (2019) performed scRNA-seq on adenovirus mediated *Brca1* knockout mammary luminal and tumor cells, and observed the continuously enhanced EMT along the tumorigenesis process 70. Hu, et al. (2021) studied the origin and evolution of breast cancers in human BRCA1 mutation carriers 4. They conducted scRNA-seq on breast cancer tissues and adjacent or prophylactic normal breast tissues from four *BRCA1* mutation carriers. Though a continuous transcriptome change was also found from normal mammary cells to tumor cells in this study, while EMT was only overt in tumorigenesis of the basal-like/ER negative tumors but not obvious in the ER high tumors. Another study from Pal, et al. (2021) performed scRNA-seq on normal, preneoplastic and tumorigenic cells of human breast, including the BRCA1 mutation carriers 71. For BRCA1-related tumorigenesis, the authors mainly focused on the immune microenvironment, which showed marked changes. Indeed, besides of the heterogeneous tumor cells, other types of cells especially the immune cells within the tumor microenvironment should also affect the intertumor heterogeneity. Further studies are required to uncover more details about the heterogeneity of the non-tumoral cells and their functions in BRCA1 related mammary tumorigenesis.

In this study, we identified and validated *Mrc2* could serve as a candidate oncogene for mammary tumorigenesis. MRC2 (also termed as CD280, ENDO180, urokinase-type plasminogen activator receptor-associated protein), encoded by *Mrc2*, is an endocytic mannose receptor. In normal mammary gland, expression of MRC2 is predominately restricted in the stromal fibroblasts, but rarely detected in the epithelia 60. But interestingly, Dirk Wienke and colleagues reported MRC2 is expressed in a small set of breast cancers, which display typical basal-like, triple negative and invasive features 61. However, in the current study we found relatively high level of *Mrc2* could also be detected in the ERα positive tumors (Table S4), and expression level of *Mrc2* is not correlated with that of *Esr1*, *Pgr* or *Erbb2* (data not shown). Notably, expression of *Mrc2* is negatively correlated with that of several epithelial markers *Cd24a*, *Cdh1*, *Epcam* and luminal markers *Krt8* and *Krt18*; meanwhile is positively correlated with that of mesenchymal/fibroblastic markers *Fn1*, *Col1a2*, *Col6a1*, *Pdgfra* and so like, in both the bulk and single cell RNA-seq data (Figure S13A). Such finding indicates the up-regulated expression of *Mrc2* may be closely associated with EMT during the tumorigenesis process. Our further gene set enrichment analysis (GSEA) of co-regulated genes of *Mrc2* indicated MRC2 may be involved in the regulation of cell cycle, PI3K-Akt/Ras/Rap1/Calcium/cGMP-PKG signaling pathways, focal adhesion, gap junction, ECM organization and other biological functions (Figure S13B-F). In our study, we found high expression of MRC2 is linked to deficiency of BRCA1, though not directly regulated by BRCA1 because transient overexpression or knockdown of BRCA1 has little effect on the expression of MRC2 (data not shown). So the increased expression of MRC2 might result from accumulated DNA damage, EMT, or other mechanisms related to BRCA1 deficiency, which needs further study to clarify.

Apart from *Mrc2*, we identified some other DEGs between the BRCA1 deficient luminal cells and tumor cells. The differential expression of these genes may promote the tumorigenesis, or functions as feedback to attempt to suppress the tumor formation. Meanwhile, we noticed that knockout of some of the DEGs did not change the proliferation of *Brca1* wild type or mutant mammary cells (Figure 7A-B). As a versatile protein, BRCA1 plays multiple roles in mammary development and tumorigenesis. So, it is interesting to further investigate the crosstalk between BRCA1 and the DEGs, and to figure out the functions and mechanisms of the DEGs in BRCA1 related mammary tumorigenesis.

In summary, our current study deciphered the inter- and intra-tumor heterogeneity of BRCA1 deficient mouse mammary tumors, and described the landscape of the mammary tumorigenesis induced by loss of BRCA1, which provides a useful resource for better understanding of BRCA1 deficiency induced mammary tumorigenesis. Besides, we identified some marker genes for BRCA1 deficient mammary tumors, which may serve as candidate targets for diagnosis, prognosis and/or treatment of BRCA1 associated breast cancers.

## Supplementary Material

Supplementary figures and tables.

Supplementary table 1.

Supplementary table 2.

Supplementary table 3.

Supplementary table 4.

Supplementary table 5.

Supplementary table 6.

Supplementary table 7.

Supplementary table 8.

Supplementary table 9.

Supplementary table 10.

Supplementary table 11.

## Abbreviations

CPTAC: Clinical Proteomic Tumor Analysis Consortium
CNVs: copy number variations
DEGs: differentially expressed genes
DDR: DNA damage response
ductal NOS: ductal carcinoma: not otherwise specified
EMT: epithelial-to-mesenchymal transition
FACS: fluorescence-activated cell sorting
GO: gene ontology
GSEA: gene set enrichment analysis
MG: mammary gland
MaSC: mammary stem cell
MT: mammary tumor
MC: medullary carcinoma
MET: mesenchymal to epithelial transition
P12: pregnant at day 12.5
PCA: principle component analysis
SNN: shared-nearest-neighbor
scRNA-seq: single cell RNA sequencing
t-SNE: t-distributed Stochastic Neighbor Embedding
TNBC: triple negative breast cancer
