# Supplementary material for: Dissecting the heterogeneity and tumorigenesis of BRCA1 deficient mammary tumors via single cell RNA sequencing
Source: Theranostics. 2021 Oct 25;11(20):9967–87. doi: 10.7150/thno.63995 (PMC8581428; doi:10.7150/thno.63995)
Supplement: Supplementary file 1 — Supplementary figures and tables. [file thnov11p9967s1.pdf]

Figure. S1

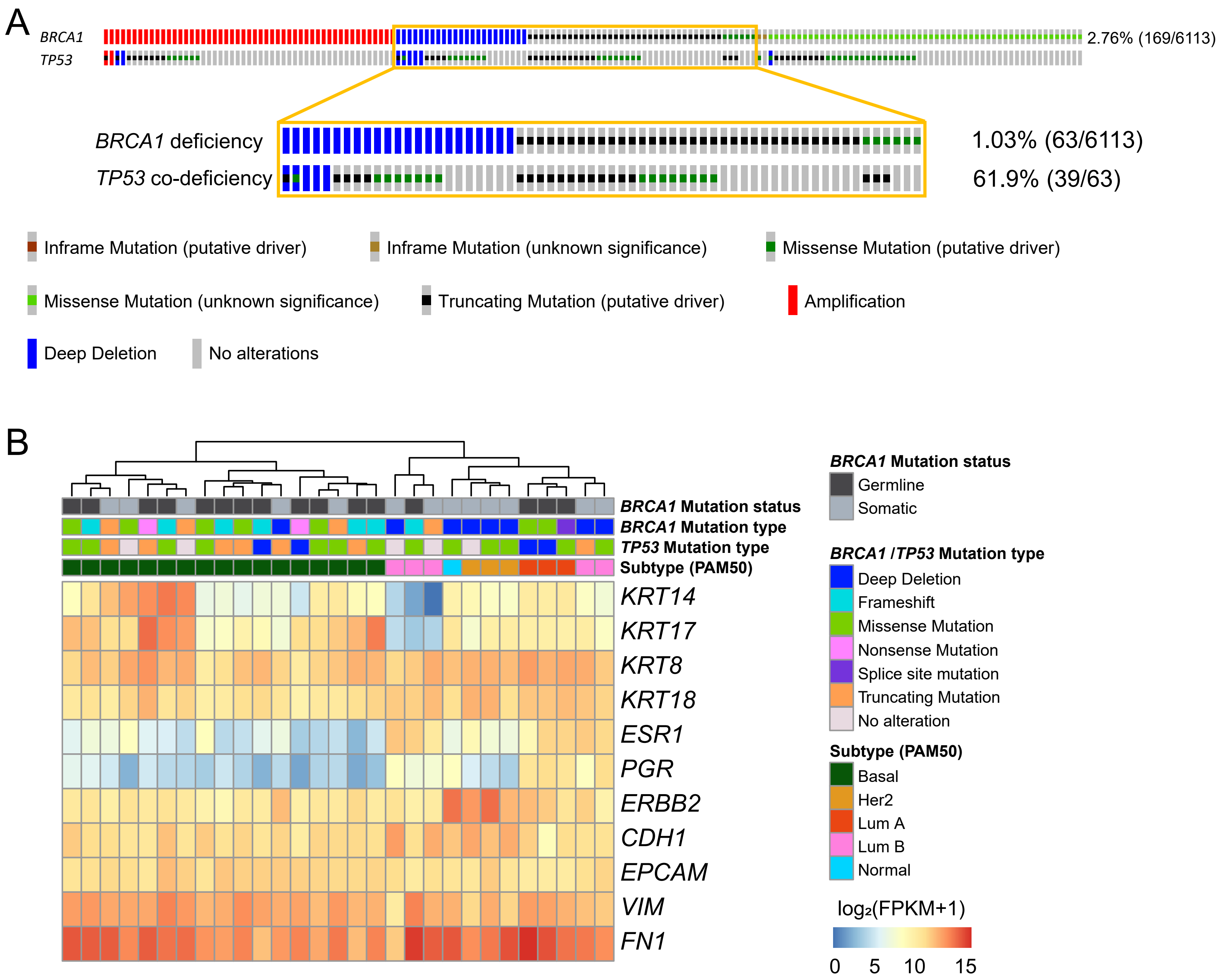

A
BRCA1
TP53
2.76% (169/6113)
BRCA1 deficiency
TP53 co-deficiency
1.03% (63/6113)
61.9% (39/63)
Inframe Mutation (putative driver)
Inframe Mutation (unknown significance)
Missense Mutation (putative driver)
Missense Mutation (unknown significance)
Truncating Mutation (putative driver)
Amplification
Deep Deletion
No alterations
B
BRCA1 Mutation status
BRCA1 Mutation type
TP53 Mutation type
Subtype (PAM50)
KRT14
KRT17
KRT8
KRT18
ESR1
PGR
ERBB2
CDH1
EPCAM
VIM
FN1
BRCA1 Mutation status
Germline
Somatic
BRCA1 / TP53 Mutation type
Deep Deletion
Frameshift
Missense Mutation
Nonsense Mutation
Splice site mutation
Truncating Mutation
No alteration
Subtype (PAM50)
Basal
Her2
Lum A
Lum B
Normal
log2(FPKM+1)
0
5
10
15

Figure S1. Intertumor heterogeneity of BRCA1-related human breast cancers from public databases. (A) A diagram summarizes the human breast cancer cases with genetic alterations of *BRCA1*. The individual samples are labeled according to the alteration types. (B) The breast cancer samples with germline or somatic *BRCA1* mutations from TCGA are hierarchically clustered. The status of *BRCA1 and TP53* (Table S3), intrinsic cancer subtype based on PAM50, and expression levels of several selected genes are shown.

Figure. S2

A

	BrT (10)	Br53T (13)	All (23)
ERalpha^{-}	8 (80.0%)	10 (76.9%)	18 (78.2%)
PR^{-}	0 (0.0%)	3 (23.1%)	20 (87.0%)
ERBB2^{-}	3 (30.0%)	6 (46.2%)	9 (39.1%)
ERalpha^{-}PR^{-}	8 (80.0%)	10 (76.9%)	18 (78.2%)
TN	2 (20.0%)	6 (46.2%)	8 (34.8%)

B

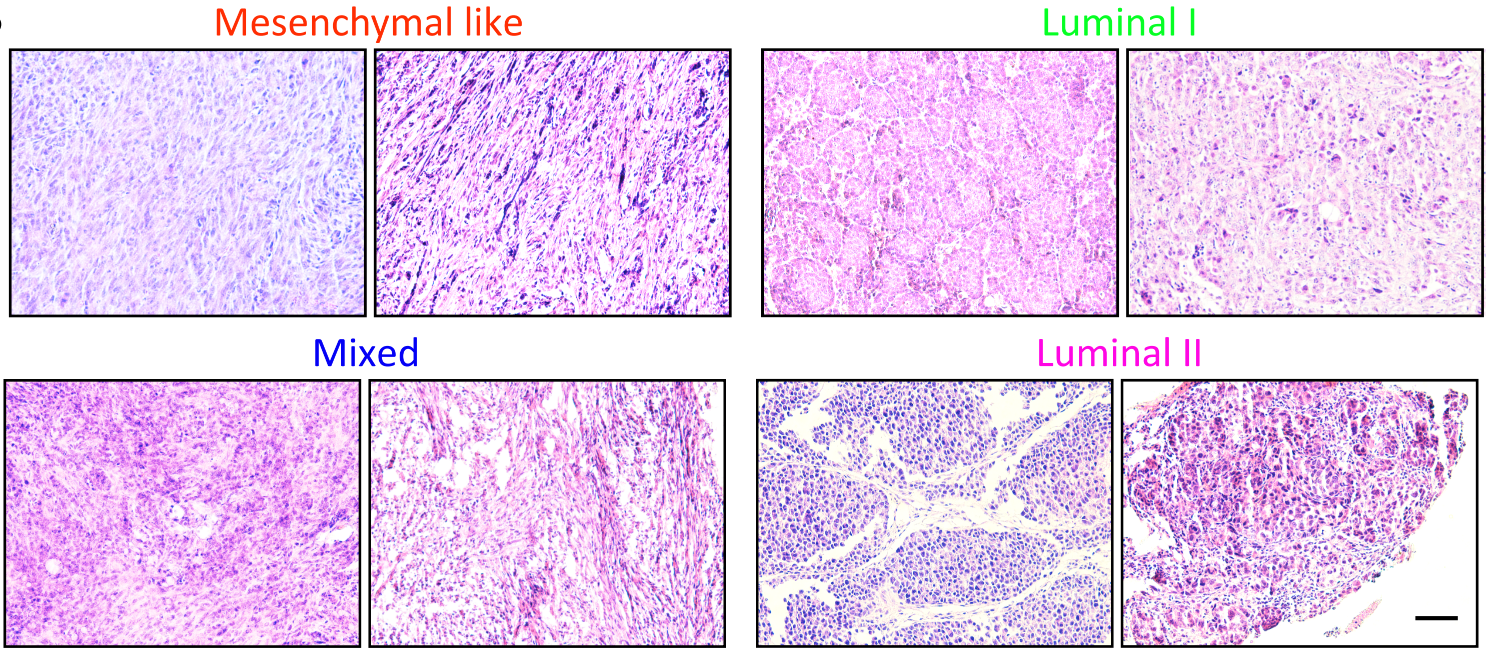

Figure S2. BRCA1-defecient mouse mammary tumors highly resemble BRCA1-related human breast cancers. (A) Summary of the ERalpha /PR /ERBB2 expression patterns (IHC) of 23 BRCA1-defecient mouse mammary tumors. (B) The mammary tumors from different groups appear respective histological features. H&E stained sections, scale bar, 50μM.

Figure. S3

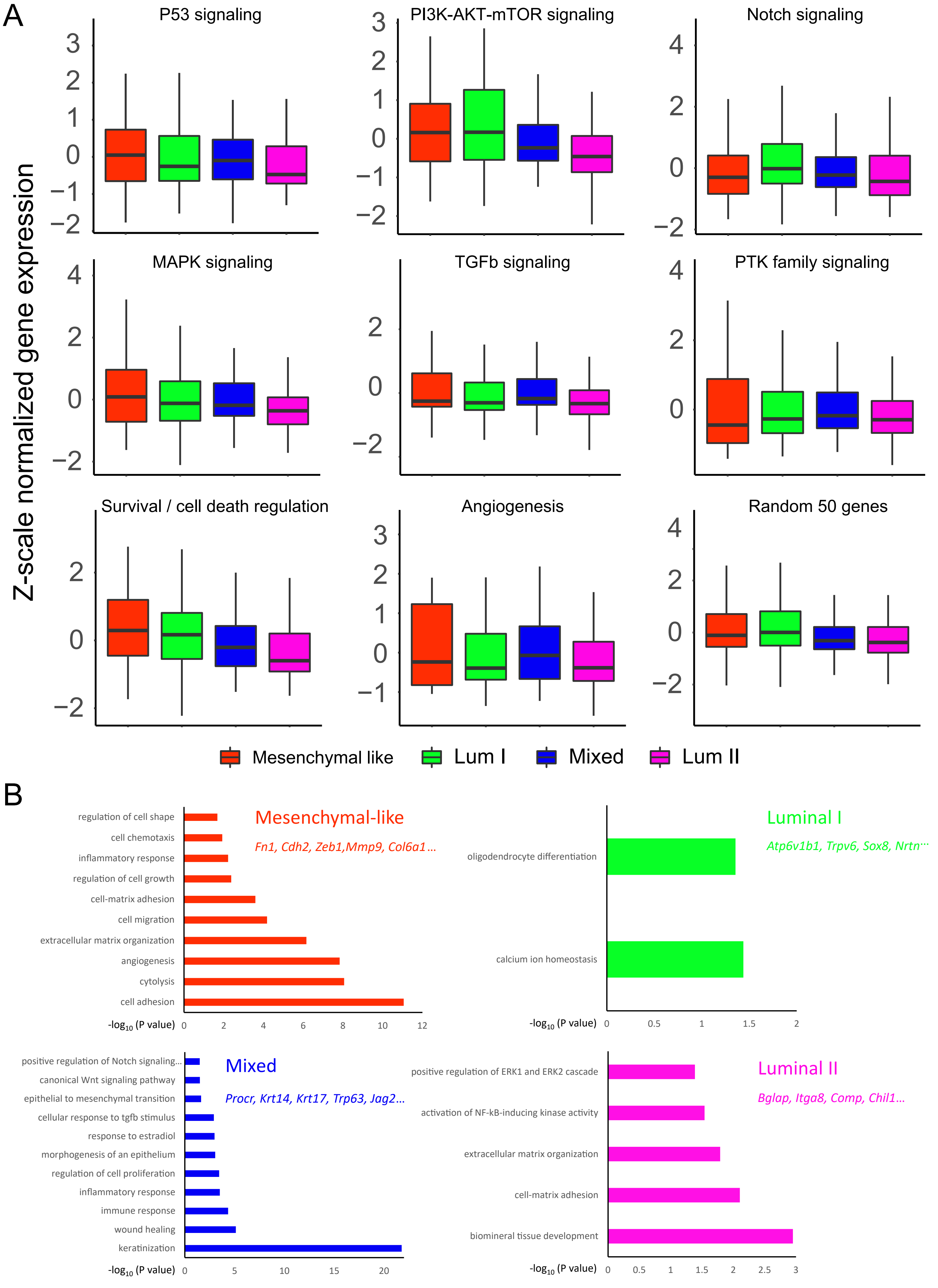
A
P53 signaling
PI3K-AKT-mTOR signaling
Notch signaling
MAPK signaling
TGFb signaling
PTK family signaling
Survival / cell death regulation
Angiogenesis
Random 50 genes
Z-scale normalized gene expression
Mesenchymal like
Lum I
Mixed
Lum II
B
Mesenchymal-like
Fn1, Cdh2, Zeb1,Mmp9, Col6a1…
regulation of cell shape
cell chemotaxis
inflammatory response
regulation of cell growth
cell-matrix adhesion
cell migration
extracellular matrix organization
angiogenesis
cytolysis
cell adhesion
-log10 (P value)
Luminal I
Atp6v1b1, Trpv6, Sox8, Nrtn…
oligodendrocyte differentiation
calcium ion homeostasis
Mixed
Procr, Krt14, Krt17, Trp63, Jag2…
positive regulation of Notch signaling…
canonical Wnt signaling pathway
epithelial to mesenchymal transition
cellular response to tgfb stimulus
response to estradiol
morphogenesis of an epithelium
regulation of cell proliferation
inflammatory response
immune response
wound healing
keratinization
Luminal II
Bglap, Itga8, Comp, Chil1…
positive regulation of ERK1 and ERK2 cascade
activation of NF-kB-inducing kinase activity
extracellular matrix organization
cell-matrix adhesion
biomineral tissue development

Figure S3. Molecular features of 4 subgroups of BRCA1-deficient mammary tumors. (A) Boxplots representing Z-scale normalized gene expression values from 4 subgroups of BRCA1-defecient mammary tumors show expression levels of different groups of genes (Table S4, Figure 2). The box represents the interquartile range and the line is the median. (B) Enriched GO terms of marker genes for 4 subgroups of BRCA1-defecient mouse mammary tumors. Representative marker genes for each subgroup are also listed (Table S4).

Figure. S4

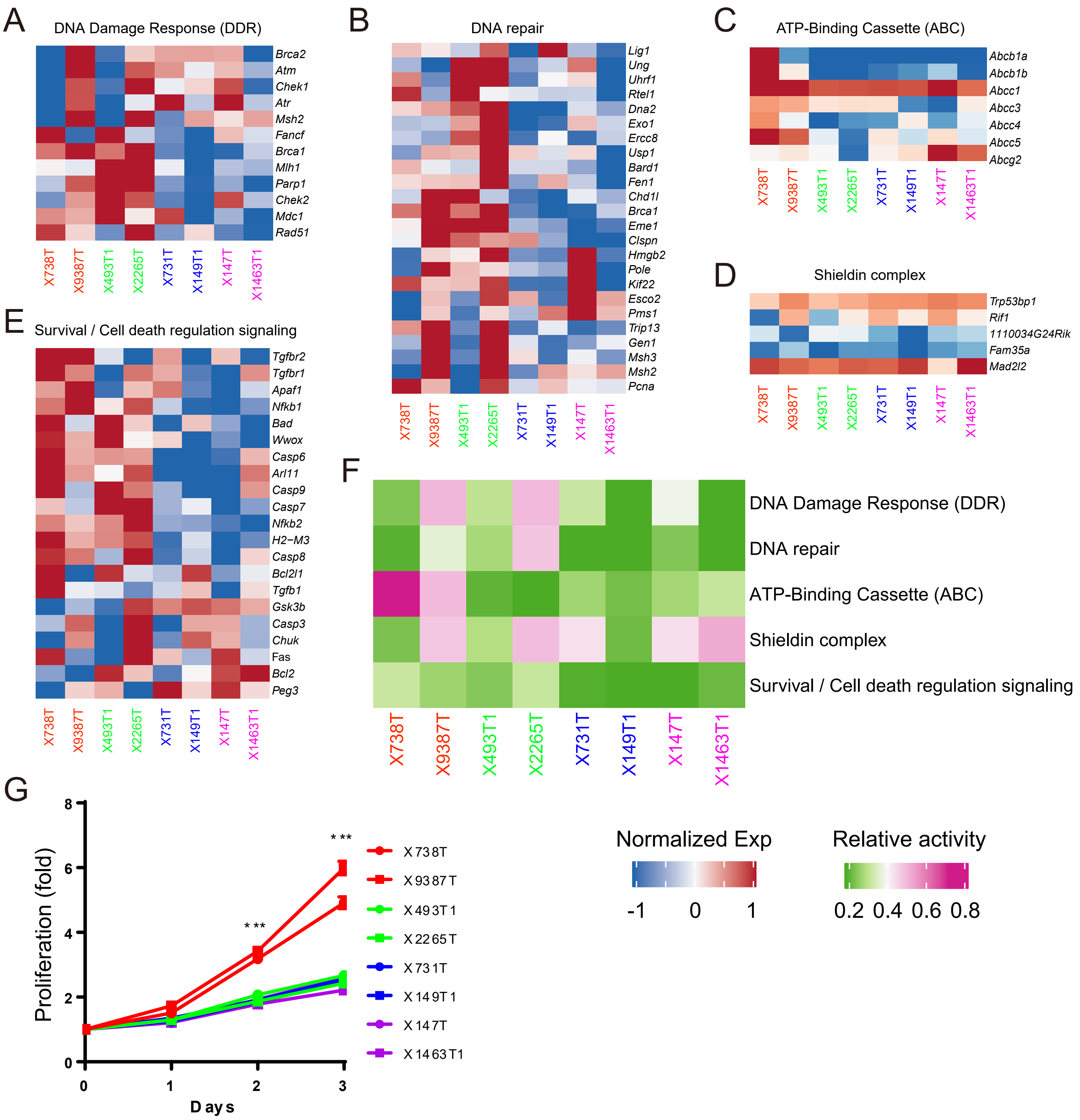

A
DNA Damage Response (DDR)
Brca2
Atm
Chek1
Atr
Msh2
Fancf
Brca1
Mlh1
Parp1
Chek2
Mdc1
Rad51
X738T
X9387T
X493T1
X2265T
X731T
X149T1
X147T
X1463T1
B
DNA repair
Lig1
Ung
Uhrf1
Rtel1
Dna2
Exo1
Ercc8
Usp1
Bard1
Fen1
Chd1l
Brca1
Eme1
Clspn
Hmgb2
Pole
Kif22
Esco2
Pms1
Trip13
Gen1
Msh3
Msh2
Pcna
C
ATP-Binding Cassette (ABC)
Abcb1a
Abcb1b
Abcc1
Abcc3
Abcc4
Abcc5
Abcg2
D
Shieldin complex
Trp53bp1
Rif1
1110034G24Rik
Fam35a
Mad2l2
E
Survival / Cell death regulation signaling
Tgfbr2
Tgfbr1
Apaf1
Nfkb1
Bad
Wwox
Casp6
Arl11
Casp9
Casp7
Nfkb2
H2−M3
Casp8
Bcl2l1
Tgfb1
Gsk3b
Casp3
Chuk
Fas
Bcl2
Peg3
F
DNA Damage Response (DDR)
DNA repair
ATP-Binding Cassette (ABC)
Shieldin complex
Survival / Cell death regulation signaling
G
Proliferation (fold)
Days
0
1
2
3
0
2
4
6
8
***
***
X738T
X9387T
X493T1
X2265T
X731T
X149T1
X147T
X1463T1
Normalized Exp
-1
0
1
Relative activity
0.2
0.4
0.6
0.8

Figure S4. Mesenchymal-like tumors' high sensitivity to cisplatin and olaparib might be due to their fast proliferation. (A -E) the heatmaps of expression levels of genes related to DNA damage response (A), DNA repair (B), ATP-binding cassette (ABC) (C), shieldin complex (D), and survival/cell death regulation signaling (E) among different tumors. (F) heatmap shows the activities of multidrug resistance related pathways (A-E) among different tumors. The pathway activities were calculated by quantification of the gene expression levels within the designated pathways. (G) cell growth rates of BRCA1-deficient tumor cell lines measured by alamarBlue assay.

Figure. S5

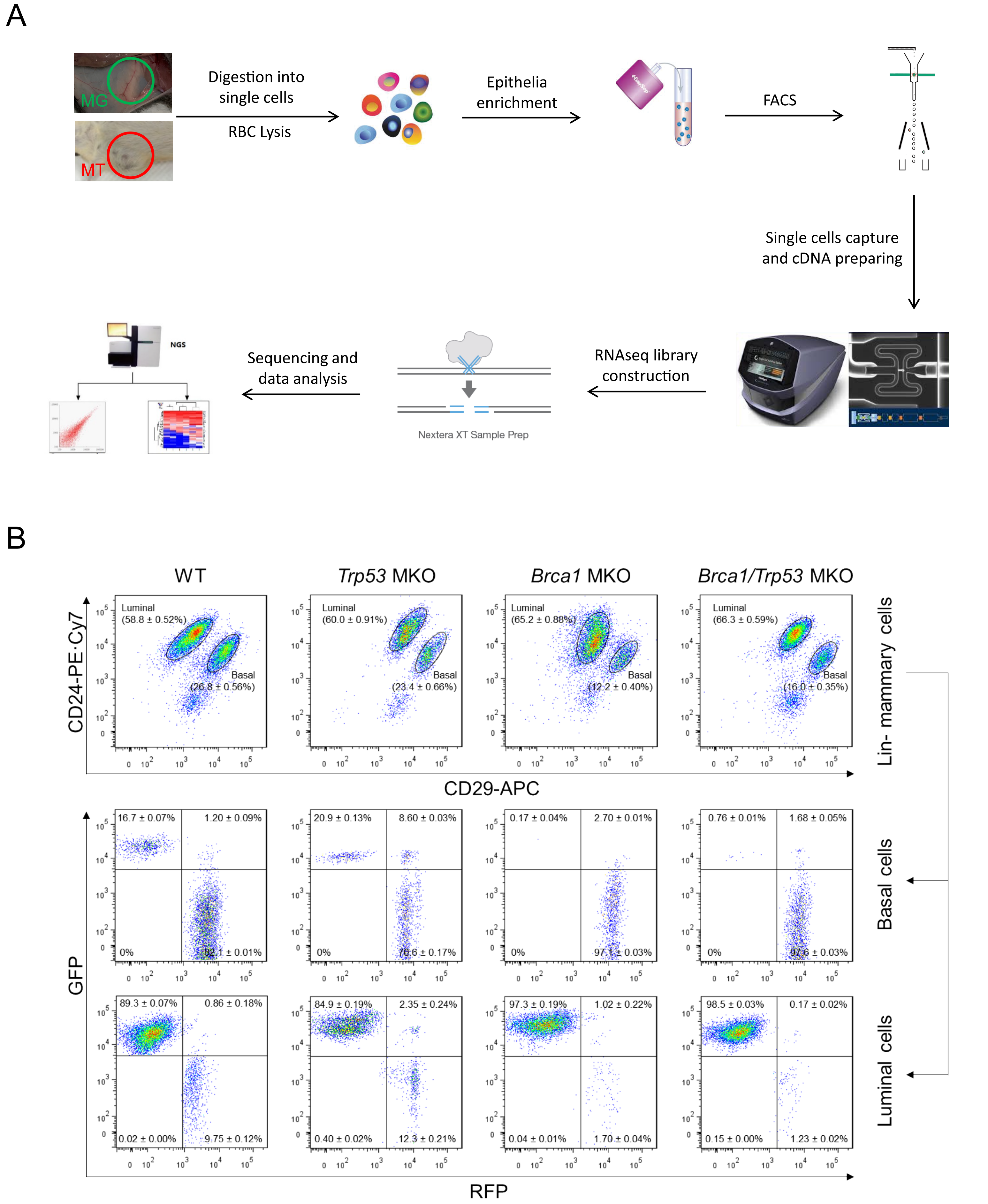
A
MG
MT
Digestion into single cells
RBC Lysis
Epithelia enrichment
FACS
Single cells capture and cDNA preparing
RNAseq library construction
Nextera XT Sample Prep
Sequencing and data analysis
NGS
B
WT
Trp53 MKO
Brca1 MKO
Brca1/Trp53 MKO
CD24-PE·Cy7
CD29-APC
Luminal (58.8 ± 0.52%)
Basal (26.8 ± 0.56%)
Luminal (60.0 ± 0.91%)
Basal (23.4 ± 0.66%)
Luminal (65.2 ± 0.88%)
Basal (12.2 ± 0.40%)
Luminal (66.3 ± 0.59%)
Basal (16.0 ± 0.35%)
Lin- mammary cells
Basal cells
Luminal cells
GFP
RFP
16.7 ± 0.07%
1.20 ± 0.09%
0%
82.1 ± 0.01%
20.9 ± 0.13%
8.60 ± 0.03%
0%
70.6 ± 0.17%
0.17 ± 0.04%
2.70 ± 0.01%
0%
97.1 ± 0.03%
0.76 ± 0.01%
1.68 ± 0.05%
0%
97.6 ± 0.03%
89.3 ± 0.07%
0.86 ± 0.18%
0.02 ± 0.00%
9.75 ± 0.12%
84.9 ± 0.19%
2.35 ± 0.24%
0.40 ± 0.02%
12.3 ± 0.21%
97.3 ± 0.19%
1.02 ± 0.22%
0.04 ± 0.01%
1.70 ± 0.04%
98.5 ± 0.03%
0.17 ± 0.02%
0.15 ± 0.00%
1.23 ± 0.02%

Figure S5. Workflow and libraries summary of the Fluidigm C1 platform based single cell RNA sequencing and flow cytometry analysis of mammary cells with different genotypes. (A) Schematic illustration of the workflow. The 4th pairs of mouse mammary glands or mammary tumors were digested and enriched for single epithelial cells. Luminal cells or tumor cells were sorted out and captured on Fluidigm C1 chips for RNA-seq libraries preparation. Then the libraries were constructed, sequenced and the data were analyzed. (B) Flow cytometry analysis of mammary cells from three-month-old female mice of different genotypes. WT, *MMTV-Cre*;*Rosa*$^{mT/mG}$; *Trp53* MKO, *MMTV-Cre*;*Trp53*$^{fl/fl}$;*Rosa*$^{mT/mG}$; *Brca1* MKO, *MMTV-Cre*; *Brca1*$^{fl/fl}$;*Rosa*$^{mT/mG}$; *Brca1/Trp53* MKO, *MMTV-Cre*; *Brca1*$^{fl/fl}$;*Trp53*$^{fl/fl}$;*Rosa*$^{mT/mG}$. For each genotype, 3 mice were sacrificed for the analysis. The cell proportions are shown as mean ± SEM.

Figure. S6

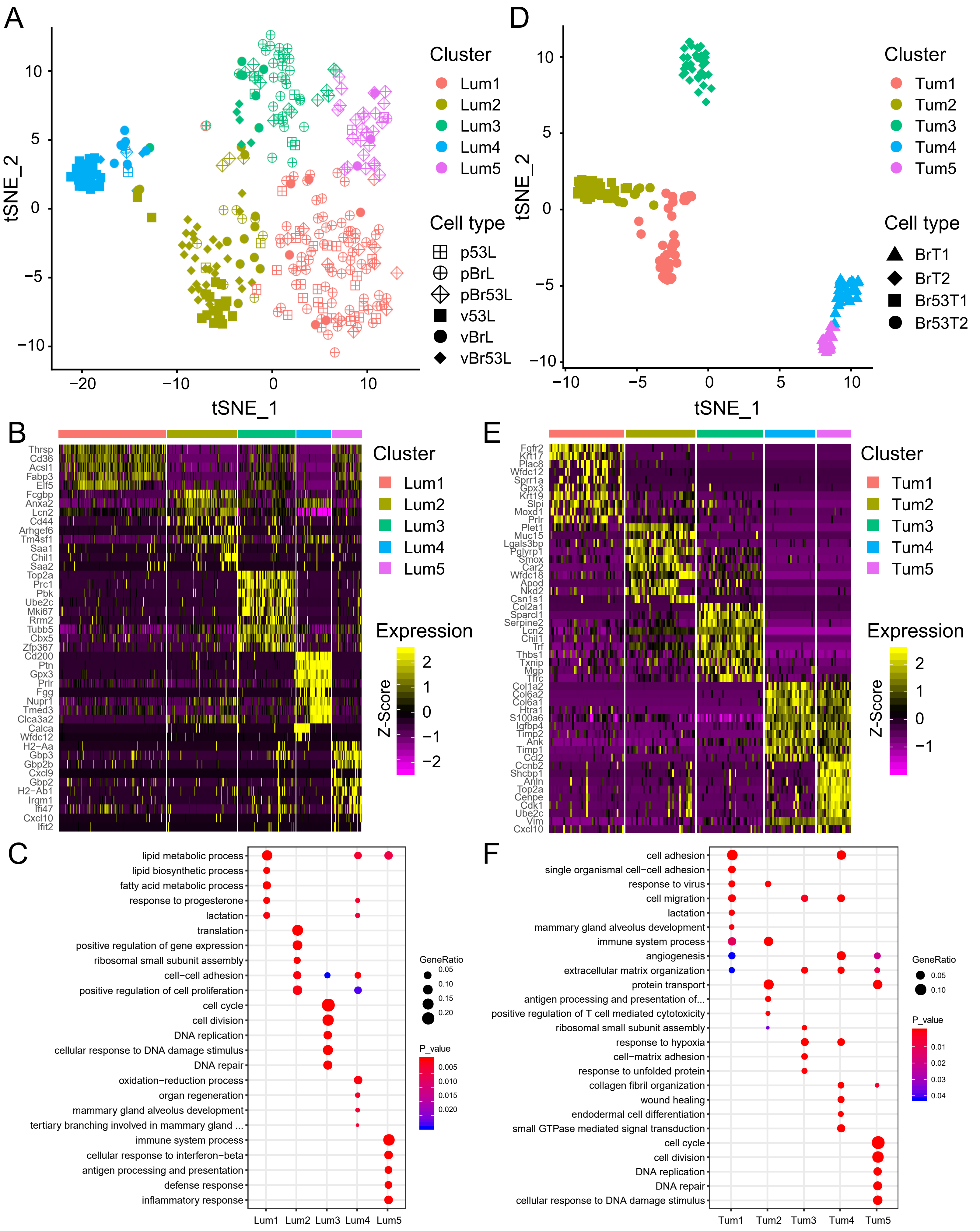
A
tSNE_1
tSNE_2
Cluster
Lum1
Lum2
Lum3
Lum4
Lum5
Cell type
p53L
pBrL
pBr53L
v53L
vBrL
vBr53L
B
Thrsp
Cd36
Acsl1
Fabp3
Elf5
Fcgbp
Anxa2
Lcn2
Cd44
Arhgef6
Tm4sf1
Saa1
Chil1
Saa2
Top2a
Prc1
Pbk
Ube2c
Mki67
Rrm2
Tubb5
Cbx5
Zfp367
Cd200
Ptn
Gpx3
Prlr
Fgg
Nupr1
Tmed3
Clca3a2
Calca
Wfdc12
H2-Aa
Gbp3
Gbp2b
Cxcl9
Gbp2
H2-Ab1
Irgm1
Ifi47
Cxcl10
Ifit2
Expression
Z-Score
C
lipid metabolic process
lipid biosynthetic process
fatty acid metabolic process
response to progesterone
lactation
translation
positive regulation of gene expression
ribosomal small subunit assembly
cell-cell adhesion
positive regulation of cell proliferation
cell cycle
cell division
DNA replication
cellular response to DNA damage stimulus
DNA repair
oxidation-reduction process
organ regeneration
mammary gland alveolus development
tertiary branching involved in mammary gland ...
immune system process
cellular response to interferon-beta
antigen processing and presentation
defense response
inflammatory response
GeneRatio
P_value
D
Tum1
Tum2
Tum3
Tum4
Tum5
BrT1
BrT2
Br53T1
Br53T2
E
Fgfr2
Krt17
Plac8
Wfdc12
Sprr1a
Gpx3
Krt19
Slpi
Moxd1
Prlr
Plet1
Muc15
Lgals3bp
Pglyrp1
Smox
Car2
Wfdc18
Apod
Nkd2
Csn1s1
Col2a1
Sparcl1
Serpine2
Lcn2
Chil1
Trf
Thbs1
Txnip
Mgp
Tfrc
Col1a2
Col6a2
Col6a1
Htra1
S100a6
Igfbp4
Timp2
Ank
Timp1
Ccl2
Ccnb2
Shcbp1
Anln
Top2a
Cenpe
Cdk1
Ube2c
Vim
Cxcl10
F
cell adhesion
single organismal cell-cell adhesion
response to virus
cell migration
lactation
mammary gland alveolus development
immune system process
angiogenesis
extracellular matrix organization
protein transport
antigen processing and presentation of...
positive regulation of T cell mediated cytotoxicity
ribosomal small subunit assembly
response to hypoxia
cell-matrix adhesion
response to unfolded protein
collagen fibril organization
wound healing
endodermal cell differentiation
small GTPase mediated signal transduction
cell cycle
cell division
DNA replication
DNA repair
cellular response to DNA damage stimulus

Figure S6. Single cell RNA sequencing of mammary luminal and tumor cells. (A and D) tSNE plots display the sub-populations of luminal cells (A) and tumor cells (D). Both the luminal and tumor cells are clustered into 5 subgroups and respectively labeled with distinct colors accordingly. The genotype and developmental stages of the mice are shown as well. (B and E) Heatmaps show the expression patterns of marker genes for each subgroup of mammary luminal cells (B) or tumor cells (E) (Table S6). (C and F) Bubble diagrams show the representatives of enriched GO terms for each subgroup of mammary luminal cells (C) or tumor cells (F).

Figure. S7

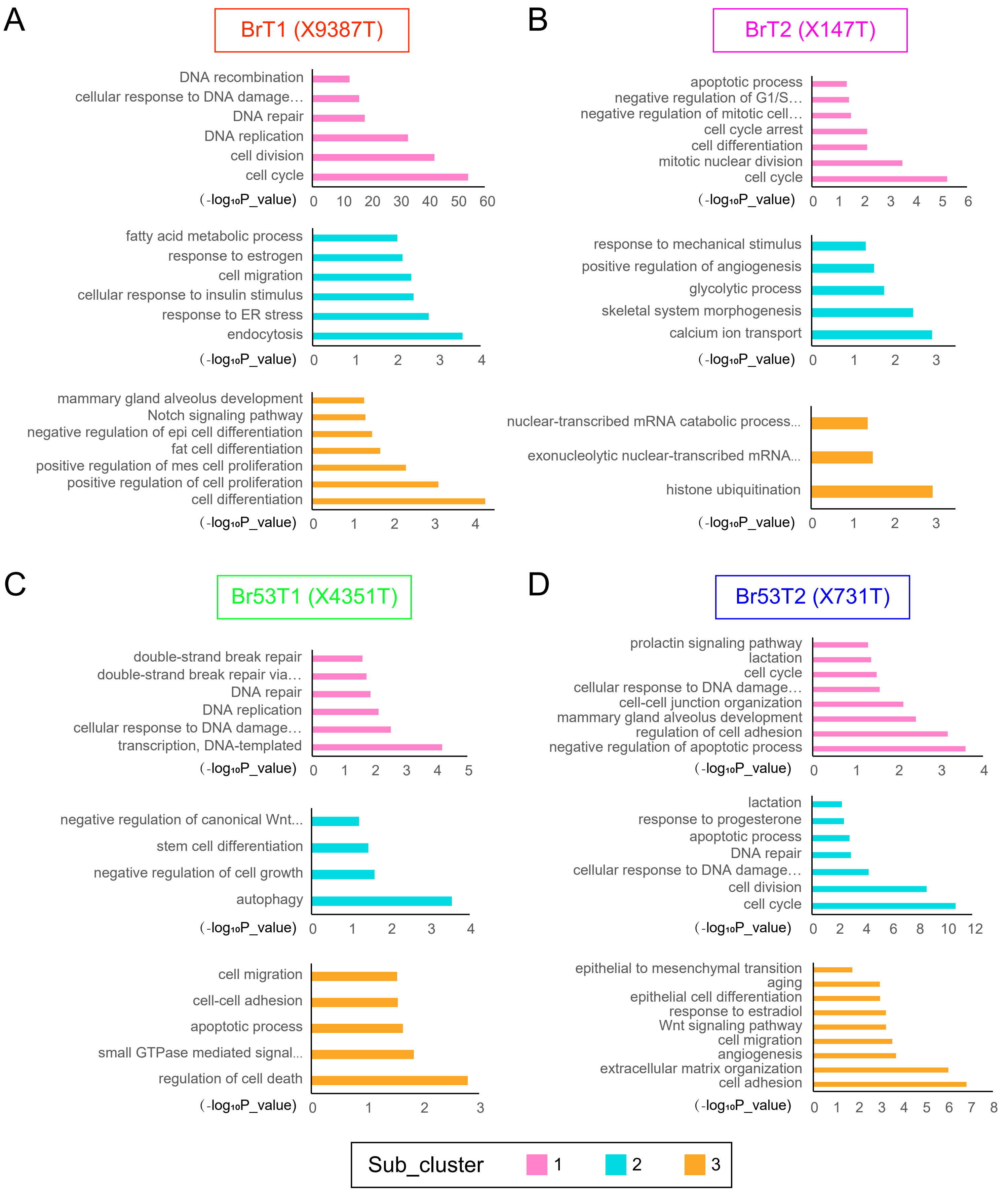
A
BrT1 (X9387T)
DNA recombination
cellular response to DNA damage…
DNA repair
DNA replication
cell division
cell cycle
(-log10P_value) 0 10 20 30 40 50 60
fatty acid metabolic process
response to estrogen
cell migration
cellular response to insulin stimulus
response to ER stress
endocytosis
(-log10P_value) 0 1 2 3 4
mammary gland alveolus development
Notch signaling pathway
negative regulation of epi cell differentiation
fat cell differentiation
positive regulation of mes cell proliferation
positive regulation of cell proliferation
cell differentiation
(-log10P_value) 0 1 2 3 4
B
BrT2 (X147T)
apoptotic process
negative regulation of G1/S…
negative regulation of mitotic cell…
cell cycle arrest
cell differentiation
mitotic nuclear division
cell cycle
(-log10P_value) 0 1 2 3 4 5 6
response to mechanical stimulus
positive regulation of angiogenesis
glycolytic process
skeletal system morphogenesis
calcium ion transport
(-log10P_value) 0 1 2 3
nuclear-transcribed mRNA catabolic process…
exonucleolytic nuclear-transcribed mRNA…
histone ubiquitination
(-log10P_value) 0 1 2 3
C
Br53T1 (X4351T)
double-strand break repair
double-strand break repair via…
DNA repair
DNA replication
cellular response to DNA damage…
transcription, DNA-templated
(-log10P_value) 0 1 2 3 4 5
negative regulation of canonical Wnt…
stem cell differentiation
negative regulation of cell growth
autophagy
(-log10P_value) 0 1 2 3 4
cell migration
cell-cell adhesion
apoptotic process
small GTPase mediated signal…
regulation of cell death
(-log10P_value) 0 1 2 3
D
Br53T2 (X731T)
prolactin signaling pathway
lactation
cell cycle
cellular response to DNA damage…
cell-cell junction organization
mammary gland alveolus development
regulation of cell adhesion
negative regulation of apoptotic process
(-log10P_value) 0 1 2 3 4
lactation
response to progesterone
apoptotic process
DNA repair
cellular response to DNA damage…
cell division
cell cycle
(-log10P_value) 0 2 4 6 8 10 12
epithelial to mesenchymal transition
aging
epithelial cell differentiation
response to estradiol
Wnt signaling pathway
cell migration
angiogenesis
extracellular matrix organization
cell adhesion
(-log10P_value) 0 1 2 3 4 5 6 7 8
Sub_cluster 1 2 3

Figure S7. Biological features of subgroups within BRCA1-defecient mouse mammary tumors. (A-D) Bar plots show the representatives of enriched GO terms for each subgroup within individual tumor. (A) for three subgroups of BrT1. (B) for three subgroups of BrT2. (C) for three subgroups of Br53T1. (D) for three subgroups of Br53T2.

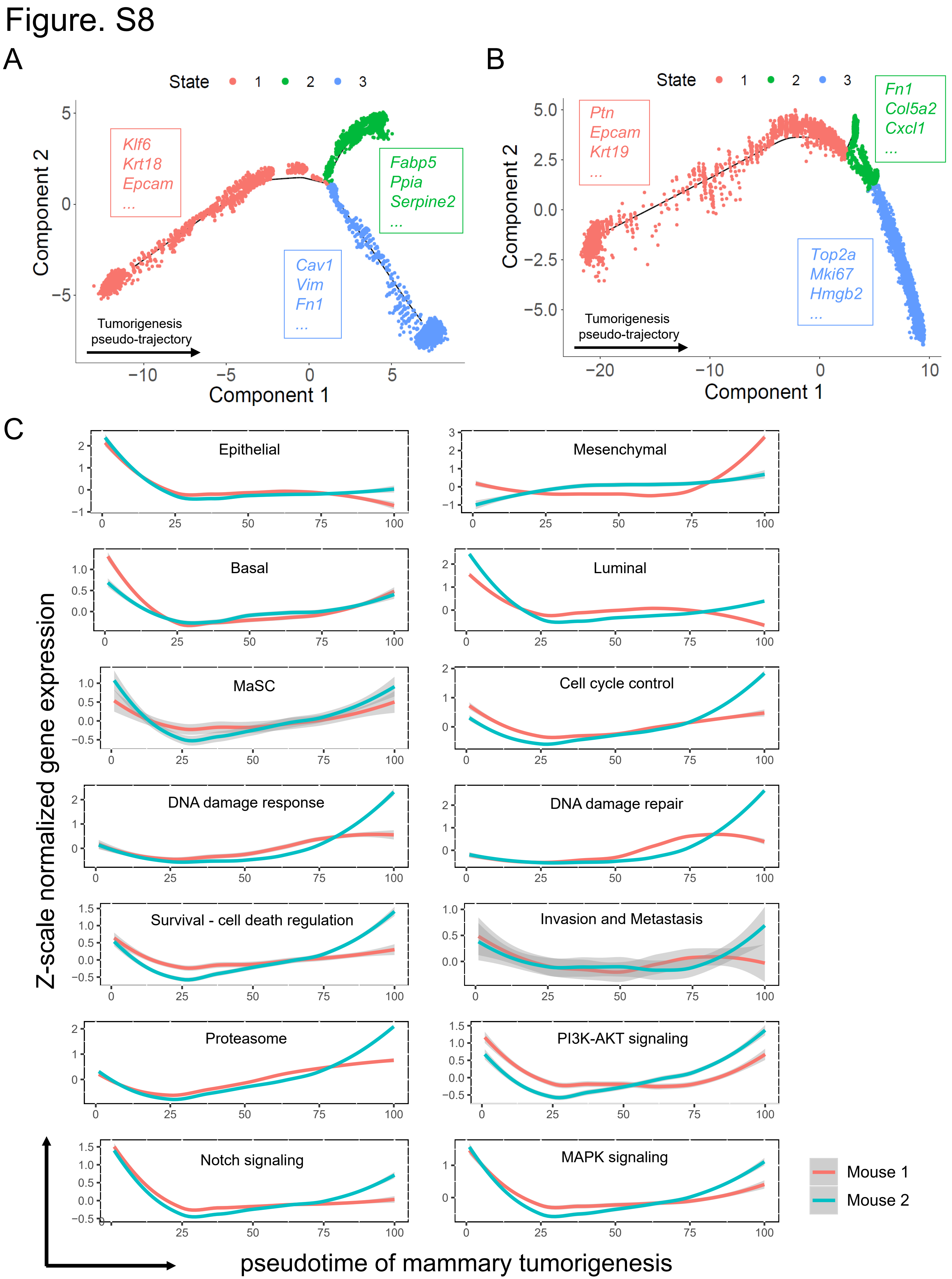
Figure. S8
A
State 1 2 3
Klf6
Krt18
Epcam
...
Fabp5
Ppia
Serpine2
...
Cav1
Vim
Fn1
...
Tumorigenesis pseudo-trajectory
Component 1
Component 2
B
State 1 2 3
Ptn
Epcam
Krt19
...
Fn1
Col5a2
Cxcl1
...
Top2a
Mki67
Hmgb2
...
Tumorigenesis pseudo-trajectory
Component 1
Component 2
C
Epithelial
Mesenchymal
Basal
Luminal
MaSC
Cell cycle control
DNA damage response
DNA damage repair
Survival - cell death regulation
Invasion and Metastasis
Proteasome
PI3K-AKT signaling
Notch signaling
MAPK signaling
Mouse 1
Mouse 2
Z-scale normalized gene expression
pseudotime of mammary tumorigenesis

Figure S8. Molecular changes during BRCA1-deficency induced tumorigenesis. (A and B) Monocle analysis reveals the pseudo-temporal trajectories of tumorigenesis in mouse 1 (A) and 2 (B). The cells are divided into three continuous states along the pseudo-temporal trajectories of tumorigenesis. Representative markers for individual state are shown as well (Table S10). (C) The variation tendency of several biological signaling pathways along the pseudo-temporal trajectory of tumorigenesis. The x-axis represents the pseudo-time of mammary tumorigenesis (from left to right) based on monocle analysis; the y-axis represents Z-scale normalized gene expression values from the genes within given gene sets (Table S4).

Figure. S9

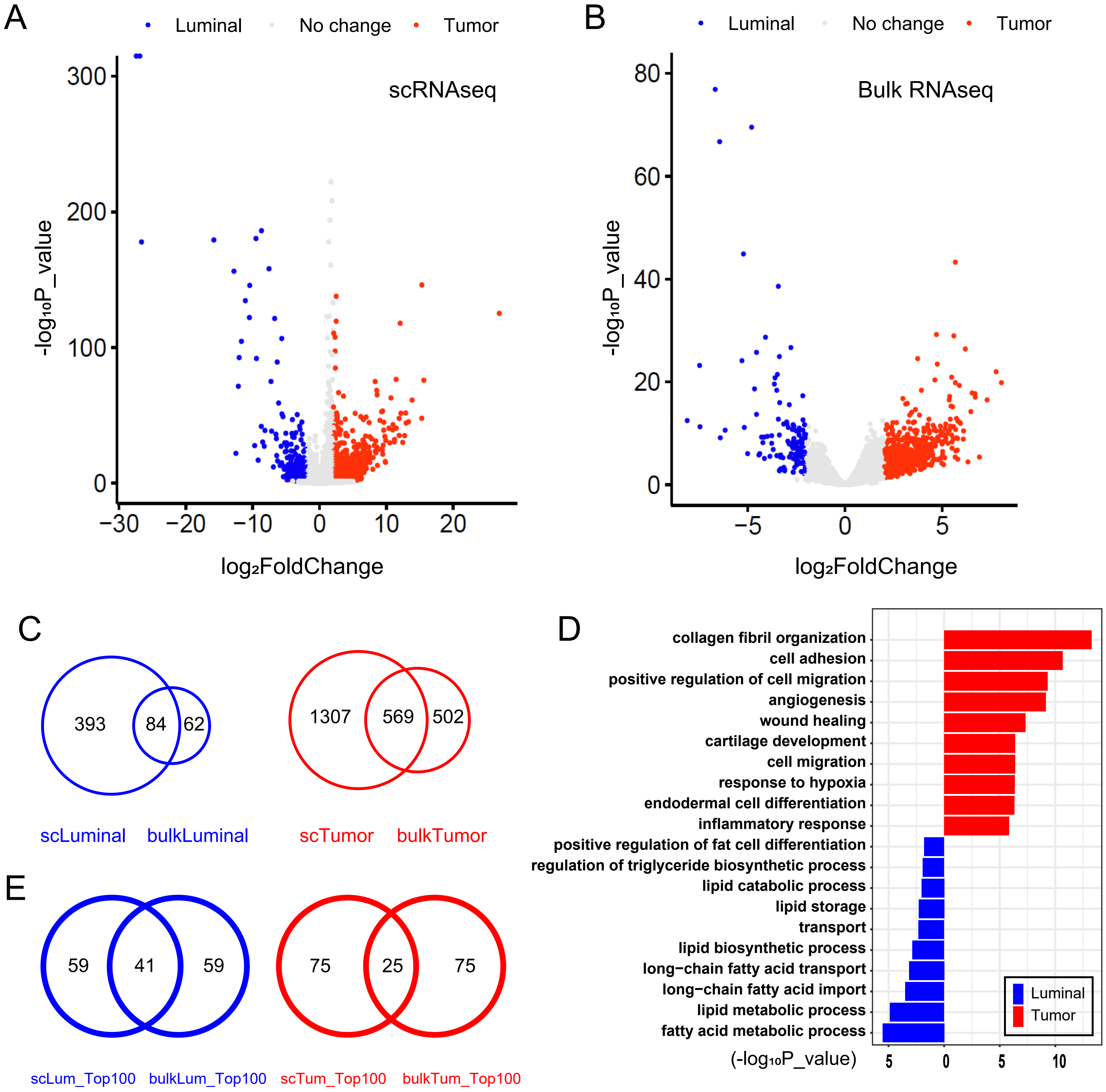

F

Fabp3	***Slc28a3***	***Acsl1***	***Cd36***	***Sectm1a***
Tmc5	***Olah***	***Chrdl2***	***Thrsp***	***Slc34a2***
Gjb6	*Cck*	*Wap*	*Rspo1*	*Muc15*
Pigr	*Csn1s2a*	*Lao1*	*Btn1a1*	*Dkkl1*
Cel	*Csn2*	*Csn1s1*	*Gjb2*	*Lpl*
Fcgbp	*Saa2*	*Bcl2l15*	*Saa1*	*Ucp3*
Sidt1	*Slc16a12*	*Gpd1*	*Zbtb16*	*Rgs8*
Timd2	*Adig*	*Hephl1*	*C130074G19Rik*	

G

Fn1	***Hmga2***	***Nkd2***	***Mrc2***	***Ank***
Cpe	***Cxcl5***	***Atp1a2***	***Aebp1***	***Crabp1***
Comp	***Sema3b***	***Loxl3***	***Fscn1***	***Plod2***
Col2a1	***Col6a2***	*Col1a2*	*Col5a1*	*Col6a1*
S100a4	*H19*	*Igfbp4*	*AA467197*	*3110079O15Rik*

Figure S9. Differentially expressed genes (DEGs) between luminal and tumor cells from analysis of single cell and bulk RNA sequencing data. (A and B) Volcano plots show the genes highly expressed in luminal cells (blue) or tumor cells (red) (Table S11). Comparison between luminal cells and tumor cells was performed by using single cell (A) or bulk (B) RNA sequencing data. (C) Venn diagrams show the common DEGs for luminal or tumor cells from single cell (A) or bulk (B) RNA sequencing data. (D) The top enriched GO terms for DEGs of luminal cells (blue) or tumor cells (red). (E) Venn diagrams show the common genes of top 100 DEGs (based on fold change) for luminal or tumor cells from single cell (A) or bulk (B) RNA sequencing data. (F and G) Genes lists of the common genes in (E). And some candidate genes were chosen for further functional analysis (labeled as black, Figure 7).

Figure. S10

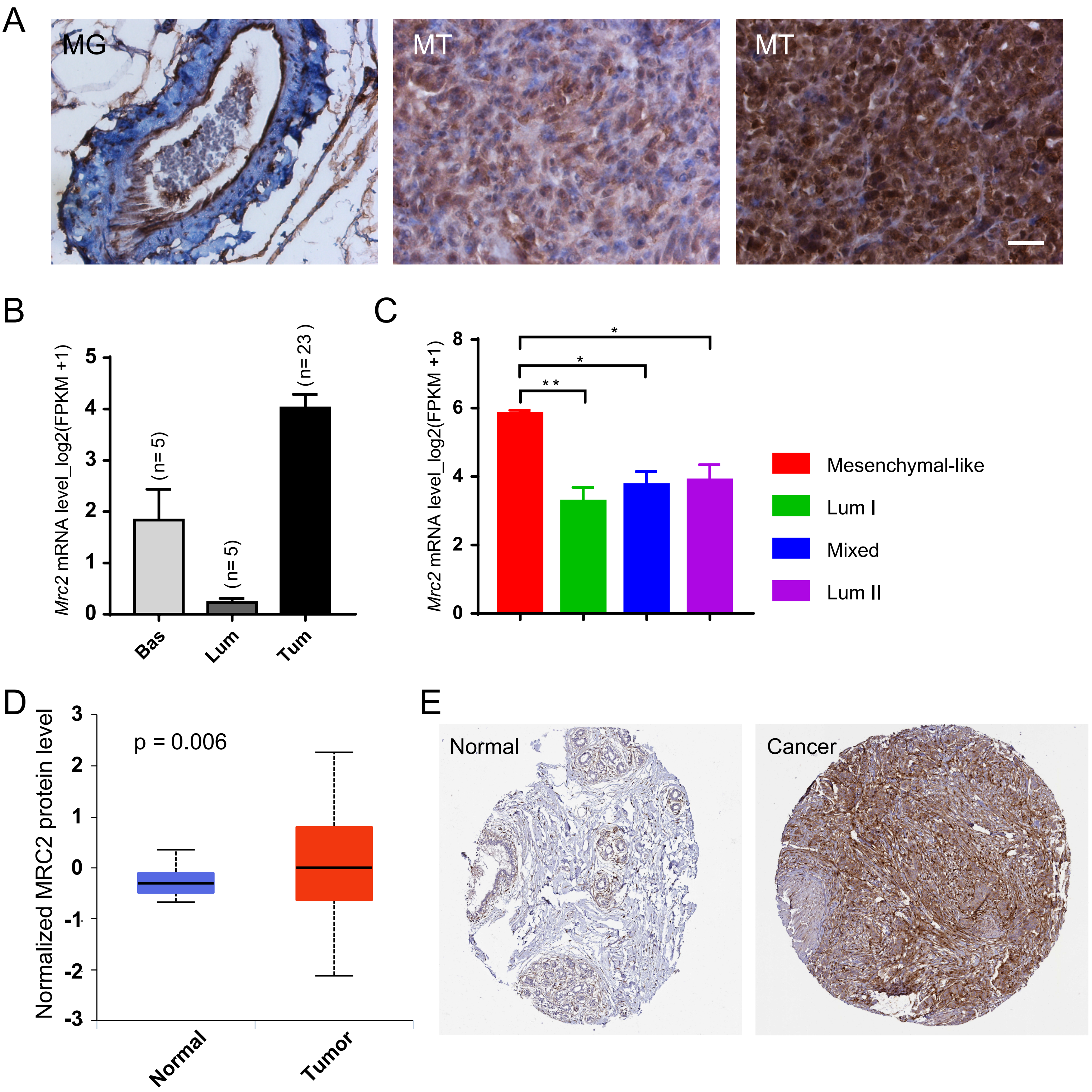
A
MG
MT
MT
B
Mrc2 mRNA level_log2(FPKM +1)
5
4
3
2
1
0
(n= 5)
(n= 5)
(n= 23)
Bas
Lum
Tum
C
Mrc2 mRNA level_log2(FPKM +1)
8
6
4
2
0
*
*
* *
Mesenchymal-like
Lum I
Mixed
Lum II
D
Normalized MRC2 protein level
3
2
1
0
-1
-2
-3
p = 0.006
Normal
Tumor
E
Normal
Cancer

Figure S10. MRC2 is highly expressed in mammary tumors and breast cancers. (A) IHC staining of MRC2 shows the expression level of MRC2 in BRCA1- deficent mouse mammary gland (MG, left) and tumors (MT, middle and right). (B) mRNA level of *Mrc2* in BRCA1-deficent mouse mammary basal cells, luminal cells and tumors. The mRNA level is quantified from RNAseq data. (C) Expression level of *Mrc2* in 4 subtypes of BRCA1-deficient mammary tumors. The mRNA level is quantified from RNAseq data. (D) Representative IHC staining pictures display the expression level of MRC2 in human normal mammary tissue (Normal, left) and breast cancer (Cancer, right). The data are cited from human protein atlas (https://www.proteinatlas.org/). (E) MRC2 is highly expressed in breast cancers compared with normal mammary tissues. The data summarizing the relative protein levels of MRC2 are collected from Clinical Proteomic Tumor Analysis Consortium (CPTAC) Confirmatory/Discovery dataset.

Figure. S11

A

Reference	A G T C T T C G A T G G C A G T G T C G T A C A C T A G G G G A
SgMrc2-1	A G T C T T C G A T G G C A G T G T C G T A - - - - - - G G G G A
SgMrc2-2	A G T C T T C A A C - - - - - - - - - - - - T A - - - - - - G G G G A
SgMrc2-3	A G T C T T C G A T G G C A G T G T C G T A C - - T A G G G G A

B

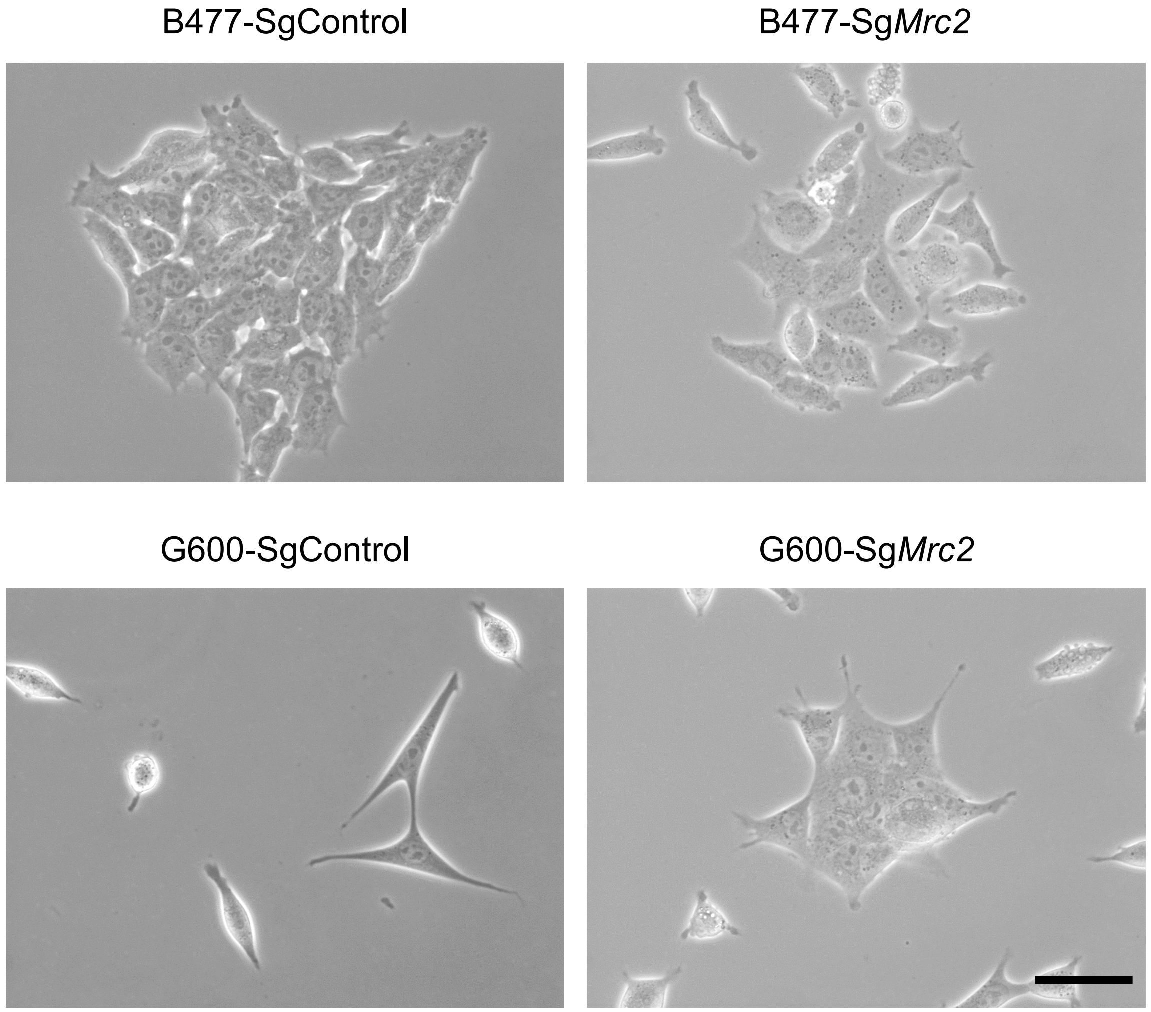

Figure S11. Knockout of *Mrc2* induces cell morphology change. (A) Sanger sequencing confirms the knockout of *Mrc2* by using CRISPR/Cas9 system. The sequence within the blue box is the sgRNA target. Three distinct clones show 5 bps, 15 bps, and 2 bps deletions around the targeting region. (B) Cell morphology changes after *Mrc2* knockout in G600 cells. While no overt change is observed after *Mrc2* knockout in B477 cells. Scale bar, 20μm.

Figure. S12

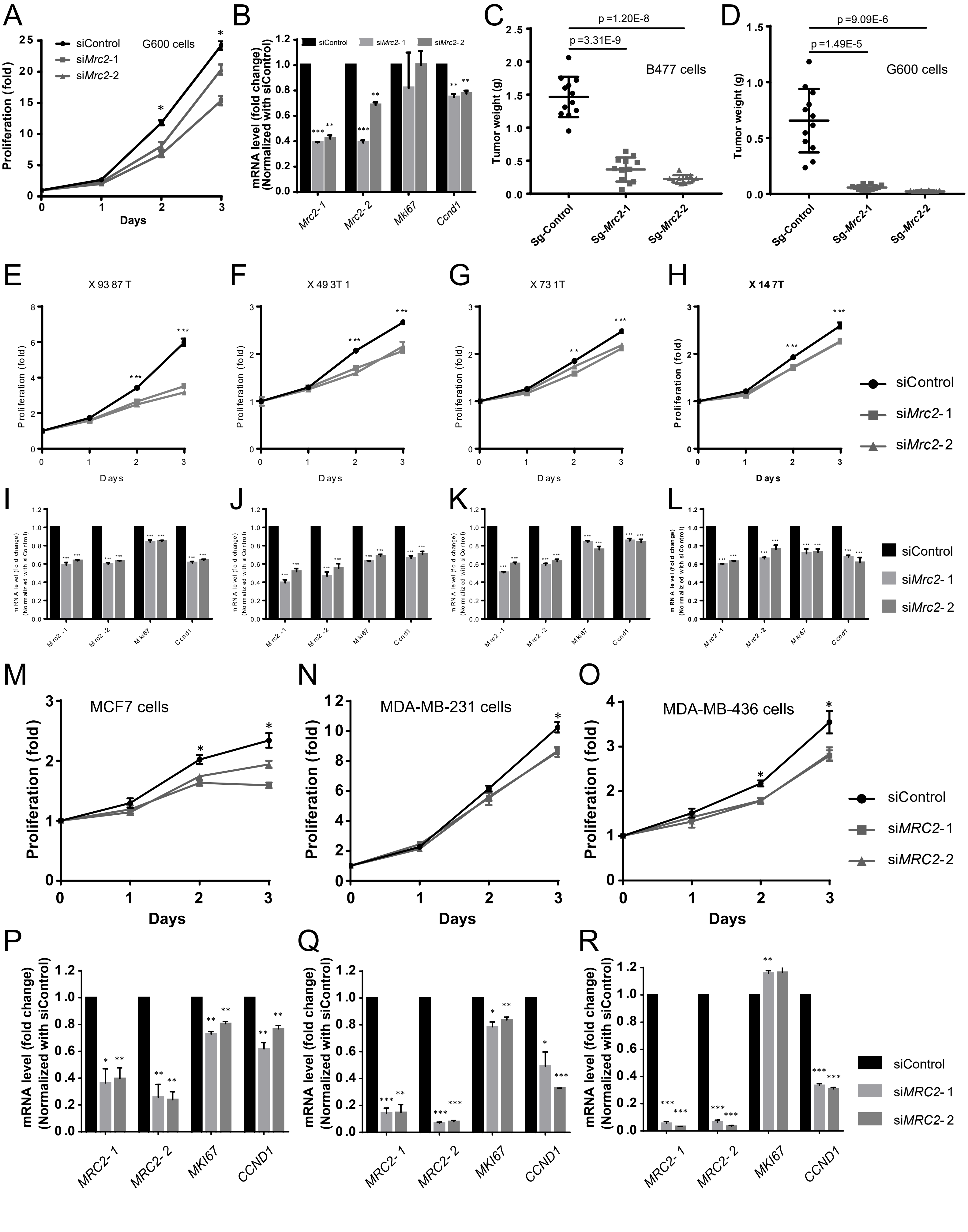
A
G600 cells
siControl
siMrc2-1
siMrc2-2
Proliferation (fold)
Days
B
siControl
siMrc2-1
siMrc2-2
mRNA level (fold change)
(Normalized with siControl)
Mrc2-1
Mrc2-2
Mki67
Ccnd1
C
p =1.20E-8
p =3.31E-9
B477 cells
Tumor weight (g)
Sg-Control
Sg-Mrc2-1
Sg-Mrc2-2
D
p =9.09E-6
p =1.49E-5
G600 cells
Tumor weight (g)
Sg-Control
Sg-Mrc2-1
Sg-Mrc2-2
E
X 93 87 T
F
X 49 3T 1
G
X 73 1T
H
X 14 7T
Proliferation (fold)
Days
siControl
siMrc2-1
siMrc2-2
I
J
K
L
siControl
siMrc2-1
siMrc2-2
M
MCF7 cells
N
MDA-MB-231 cells
O
MDA-MB-436 cells
Proliferation (fold)
Days
siControl
siMRC2-1
siMRC2-2
P
Q
R
mRNA level (fold change)
(Normalized with siControl)
MRC2-1
MRC2-2
MKI67
CCND1
siControl
siMRC2-1
siMRC2-2

Figure S12. Knockout of *Mrc2* blocked tumor cells growth *in vivo* and *in vitro*. (A) Knockdown of *Mrc2* by using siRNAs inhibits G600 cells proliferation. (B) Relative mRNA levels of *Mrc2*, *Mki67*, and *Ccnd1* after *Mrc2* knockdown in G600 cells. (C and D) Summary of tumor weights of B477 (C) or G600 (D) cells formed xenografts in nude mice. (E-H). Knockdown of *Mrc2* blocked BRCA1 mutant tumor cells growth regardless of subtypes. Tumor cell lines were derived from X9387T (E, mesenchymal like type), X493T1 (F, Lum I type), X731T (G, mixed type) and X147T (H, Lum II type). (I-L) Relative mRNA levels of *Mrc2*, *Mki67*, and *Ccnd1* after *Mrc2* knockdown in BRCA1 mutant mammary tumor cells. (M-O) Knockdown of *MRC2* by using siRNAs inhibits proliferation of MCF7, MDA-MB-231, and MDA-MB-436 cells. (P-R) Relative mRNA levels of *MRC2*, *MKI67*, and *CCND1* after *MRC2* knockdown in MCF7, MDA-MB-231, and MDA-MB-436 cells. *, p value <0.05; **, p value <0.01; ***, p value <0.001.

Figure. S13

A

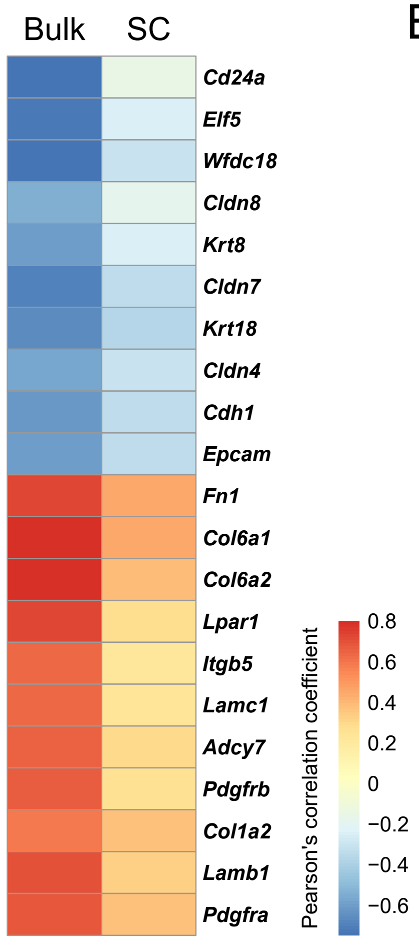

B

GSEA ID	GSEA term	Bulk-RNAseq		sc-RNAseq	
		NES#	P.adjust*	NES#	P.adjust*
mmu00010	Glycolysis / Gluconeogenesis	1.569	0.044	1.558	0.020
mmu04014	Ras signaling pathway	1.650	0.017	1.279	0.044
mmu04015	Rap1 signaling pathway	1.610	0.017	1.307	0.036
mmu04020	Calcium signaling pathway	1.983	0.017	1.394	0.020
mmu04022	cGMP-PKG signaling pathway	1.595	0.017	1.367	0.026
mmu04110	Cell cycle	1.509	0.033	1.371	0.032
mmu04142	Lysosome	2.330	0.017	1.349	0.048
mmu04151	PI3K-Akt signaling pathway	1.760	0.017	1.447	0.014
mmu04510	Focal adhesion	1.980	0.017	1.649	0.014
mmu04512	ECM-receptor interaction	1.996	0.017	1.682	0.014
mmu04540	Gap junction	1.917	0.017	1.585	0.014
mmu04912	GnRH signaling pathway	1.786	0.017	1.415	0.036
mmu04974	Protein digestion and absorption	1.921	0.017	1.756	0.014
mmu05205	Proteoglycans in cancer	1.864	0.017	1.531	0.014

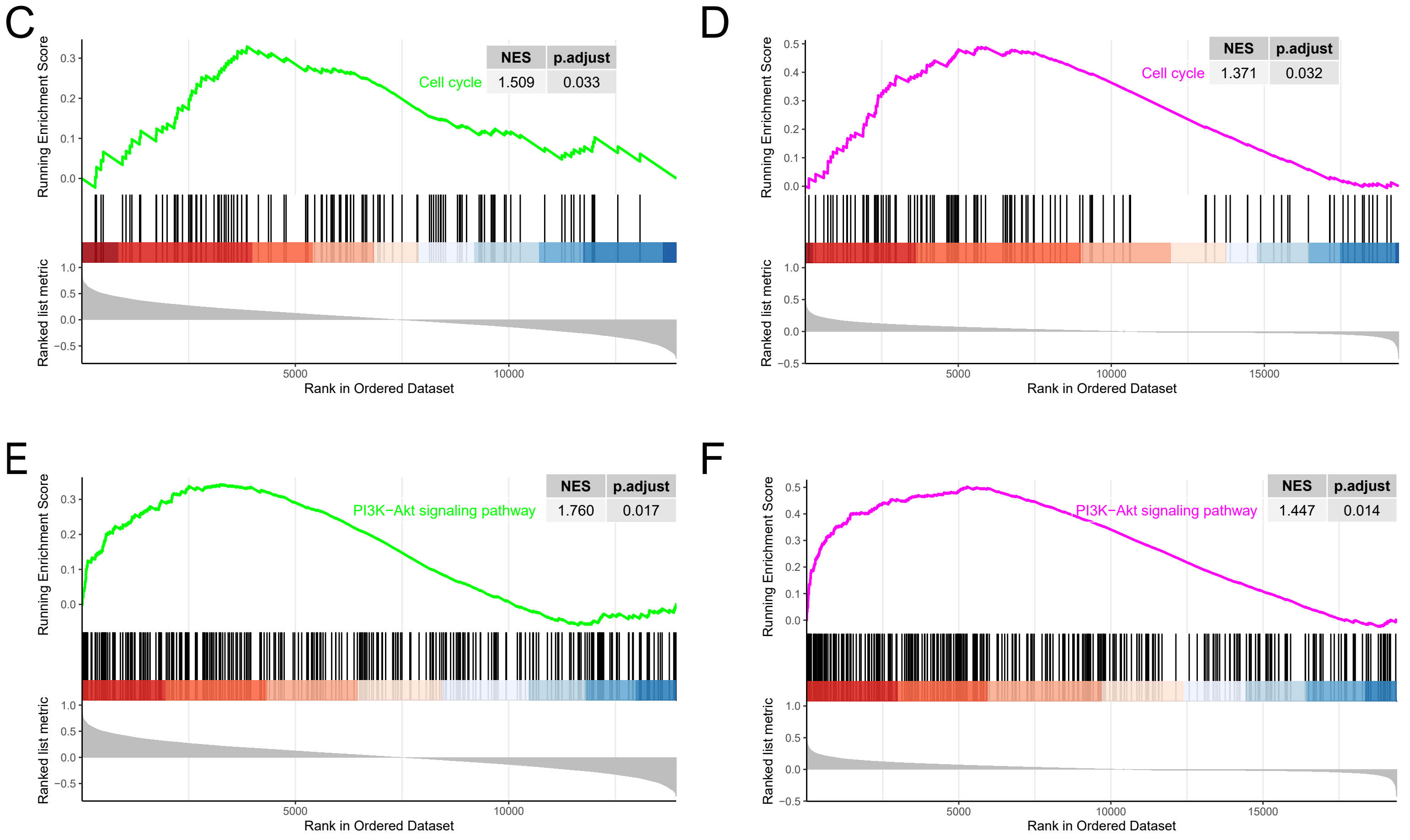

Figure S13. MRC2 is involved in regulation of cell cycle, extracellular matrix (ECM) and other pathways. (A) Heatmap shows the expression correlation coefficients of some co-regulated genes with *Mrc2*. The Pearson's correlation coefficient of mRNA level of each gene with that of *Mrc2* was calculated based on the bulk and single cell RNA-seq data (Table S4 and S5). (B) Enriched biological functions of genes co-regulated with *Mrc2* are identified by GSEA analysis. #, NES, Normalized Enrichment Score; *, P.adjust, adjusted p value. (C-F), the GSEA analysis results of cell cycle pathway (C and D) and PI3K-Akt signaling pathway (E and F) by using bulk (C and E) and single cell (D and F) RNA-seq data.
